# Indirect impact of Covid-19 on hospital care pathways in Italy

**DOI:** 10.1038/s41598-021-00982-4

**Published:** 2021-11-02

**Authors:** Teresa Spadea, Chiara Di Girolamo, Tania Landriscina, Olivia Leoni, Silvia Forni, Paola Colais, Caterina Fanizza, Alessandra Allotta, Roberta Onorati, Roberto Gnavi, the Mimico-19 working group, Teresa Spadea, Roberto Gnavi, Tania Landriscina, Roberta Onorati, Alessandro Migliardi, Giuseppe Costa, Olivia Leoni, Michele Ercolanoni, Chiara Di Girolamo, Elena Berti, Nicola Caranci, Maria Luisa Moro, Silvia Forni, Valeria Di Fabrizio, Sara D’Arienzo, Fabrizio Gemmi, Paola Colais, Luigi Pinnarelli, Mariangela D’Ovidio, Maria Balducci, Marina Davoli, Caterina Fanizza, Vito Petrarolo, Giulia Piepoli, Lucia Bisceglia, Alessandra Allotta, Achille Cernigliaro, Salvatore Scondotto

**Affiliations:** 1 Epidemiology Unit ASL TO3, Piedmont Region, Turin, Italy; 2 Regional Health and Social Care Agency of Emilia-Romagna Region, Viale Aldo Moro, 21, 40128 Bologna, Italy; 3 Regional Epidemiological Observatory, Lombardy Region, Milan, Italy; 4 Regional Health Agency of Tuscany Region, Florence, Italy; 5 Department of Epidemiology, Regional Health Service, Lazio Region, Rome, Italy; 6 Regional Healthcare Agency of Puglia Region, Bari, Italy; 7 Department of Health Services and Epidemiological Observatory, Sicily Region, Palermo, Italy

**Keywords:** Epidemiology, Health care

## Abstract

Earlier in 2020, seven Italian regions, which cover 62% of the Italian population, set up the Mimico-19 network to monitor the side effects of the restrictive measures against Covid-19 on volumes and quality of care. To this aim, we retrospectively analysed hospital discharges data, computing twelve indicators of volume and performance in three clinical areas: cardiology, oncology, and orthopaedics. Weekly indicators for the period January–July 2020 were compared with the corresponding average for 2018–2019; comparisons were performed within 3 sub-periods: pre-lockdown, lockdown, and post-lockdown. The weekly trend of hospitalisations for ST-segment elevation myocardial infarction (STEMI) showed a 40% reduction, but the proportion of STEMI patients with a primary PTCA did not significantly change from previous years. Malignant neoplasms surgery volumes differed substantially by site, with a limited reduction for lung cancer (< 20%) and greater declines (30–40%) for breast and prostate cancers. The percentage of timely surgery for femoral neck in the elderly remained constantly higher than the previous 2 years whereas hip and knee replacements fell dramatically. Hospitalisations have generally decreased, but the capacity of a timely and effective response in time-dependent pathways of care was not jeopardized throughout the period. General trends did not show important differences across regions, regardless of the different burden of Covid-19. Preventive and primary care services should adopt a pro-active approach, moving towards the identification of at-risk conditions that were neglected during the pandemic and timely addressing patients to the secondary care system.

## Introduction

In Italy, the Covid-19 pandemic has caused more than 4.2 million cases of infection and over 130,000 deaths to date^1^. In response to the first epidemic outbreak in Lombardy, which rapidly spread to other regions, the government issued the first national lockdown starting on 9 March 2020. Since then, evidence had accrued on its possible effects on people’s health^2,3^. Services whose benefits could be lower than the patient’s risk of infection and the organizational difficulties were postponed, and the population was recommended to avoid unnecessary access to health services. This indication, however, may have caused—for fear of contagion or misinterpretation of the norm—further delays in recognizing symptoms and timely accessing diagnosis and treatment, even for non-deferrable conditions. Furthermore, several specialist departments had their hospital beds cut down to face the huge flow of Covid-19 patients.

Earlier in 2020, seven Italian regions (Piedmont, Lombardy, Emilia-Romagna, Tuscany, Lazio, Puglia, and Sicily) have therefore set up the Mimico-19 network to monitor the side effects of the restrictive measures against Covid-19 on the quality of care. These seven regions total about 37 million inhabitants (62% of the Italian population) and cover areas of the country with a different epidemic burden^4^.

The objective of this report is to describe the indirect impact of the pandemic and the lockdown measures on hospital activities through indicators of volume and performance in three clinical areas: cardiology, oncology, and orthopaedics. These clinical areas were chosen because of their high volumes of activity and the severity of the conditions.

## Methods

Using data from the regional hospital discharge databases, we defined four indicators for each of the three clinical areas (the ICD-9-CM codes included in the indicators are detailed in the Supplementary Information 1):

- Cardiology: volumes of acute admissions for ST and non-ST segment elevation myocardial infarction (STEMI and NSTEMI); percentage of coronary angioplasty interventions (PTCA) in STEMI patients carried out within 90 min from the admission as an indicator of performance in a time-dependent procedure; in-hospital mortality in STEMI patients as an indicator of severity and outcome of the disease.
- Oncology: volumes of surgical interventions for all malignant neoplasms and volumes of surgical interventions for lung, breast, and prostatic cancers, which represent a usually non-deferrable surgery, a proxy indicator for screening activities, and a scheduled operation, respectively.
- Orthopaedics: volumes of hip and knee replacement, which are generally scheduled surgeries; acute admissions for femoral neck fracture in the elderly (> 65 years of age) and, among these admissions, percentage of interventions performed within 2 days, which is another time-dependent procedure indicator.

Regional indicators were pooled into national estimates. They were computed on a weekly basis for the period January–July 2020 and compared through the paired-sample Wilcoxon test with the average of the corresponding months in 2018–2019 within three sub-periods: *pre-lockdown* (until March 8), *lockdown* (March 9–May 17) and *post-lockdown* (from May 18 onwards). We also calculated the weekly percentage variation of the 2020 value versus the 2018–2019 average.

We used unidentifiable aggregated data to carry out secondary analyses on information that are already routinely collected for administrative reasons by our institutions (regional health authorities themselves), and that subsequently feed the national information system of the Ministry of Health. The set of indicators analysed are validated within the PNE National Healthcare Outcomes Programme, a national evaluation programme run by the National Agency for Regional Healthcare Services; their use is authorised for routine activities of health service research and quality improvement by regional health authorities and for which individuals' written consent is not required under the current national regulation (latest ministerial decree number 261 issued on the 07/12/2016).

Data have been processed following appropriate privacy regulations; study characteristics and results are reported according to the STROBE guidelines.

## Results

Here we report the results for the pooled indicators; detailed regional results are presented in the Supplementary Information 2 and highlighted in the text only when important variations occur.

### Cardiology

**Figure 1:**
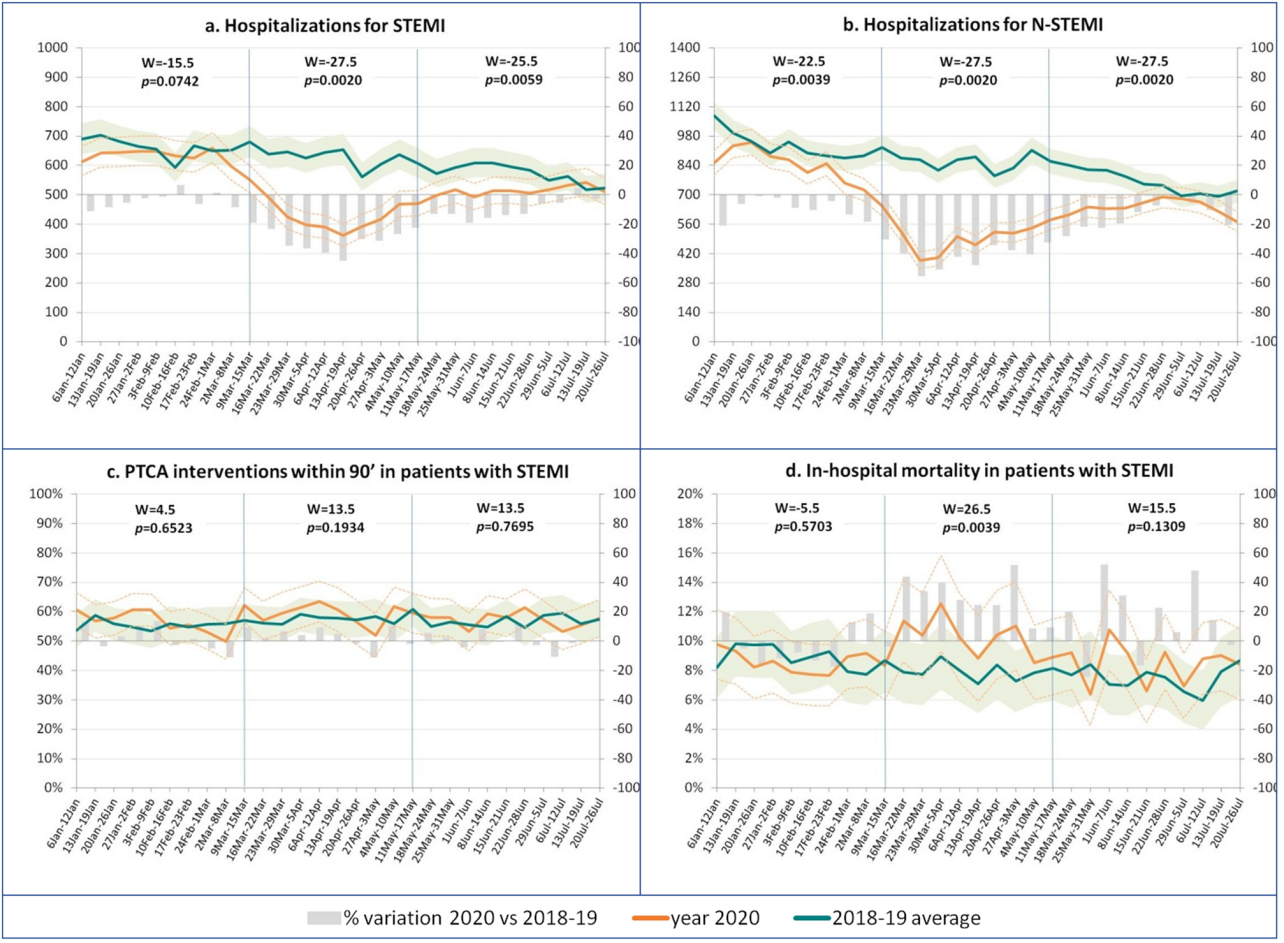
Impact of Covid-19 on cardiological hospital care in Italy: volumes and performance by sub-period*. Weekly trend of indicators (left axis) with 95% CI and % variations (right axis)—January–July 2020 versus 2018–2019 average. *Pre-lockdown, lockdown (9/3–17/5), post-lockdown—W values and *p*-values from the paired-sample Wilcoxon test for comparisons within each sub-period.

The weekly trend of hospitalizations for STEMI (Fig. 1a) showed a gradual reduction in volumes, which began in late February and peaked in mid-April (% variation about − 40%). Trends revealed a highly significant difference during lockdown, although STEMI hospitalization showed a downward tendence even before the pandemic. In the post-lockdown, volumes went slowly back to the starting values. The hospitalization for NSTEMI (Fig. 1b) showed a similar trend, but the average percent reduction was greater (over 55% at the end of March) and the post-lockdown recovery was slower. The proportion of STEMI patients with a primary PTCA within 90 min from admission (Fig. 1c), which could be calculated only in four regions, remained stable over time without significant changes from previous years. On the other hand, in-hospital mortality significantly increased by 26% on average during lockdown and by 15% in the subsequent period (Fig. 1d). This unfavourable outcome was almost entirely driven by Lombardy, the region with the earliest and greatest burden of Covid-19; all the other regions showed non-significant differences in mortality, although with large weekly fluctuations (Fig. S1d, Supplementary Information 2).

### Oncology

**Figure 2:**
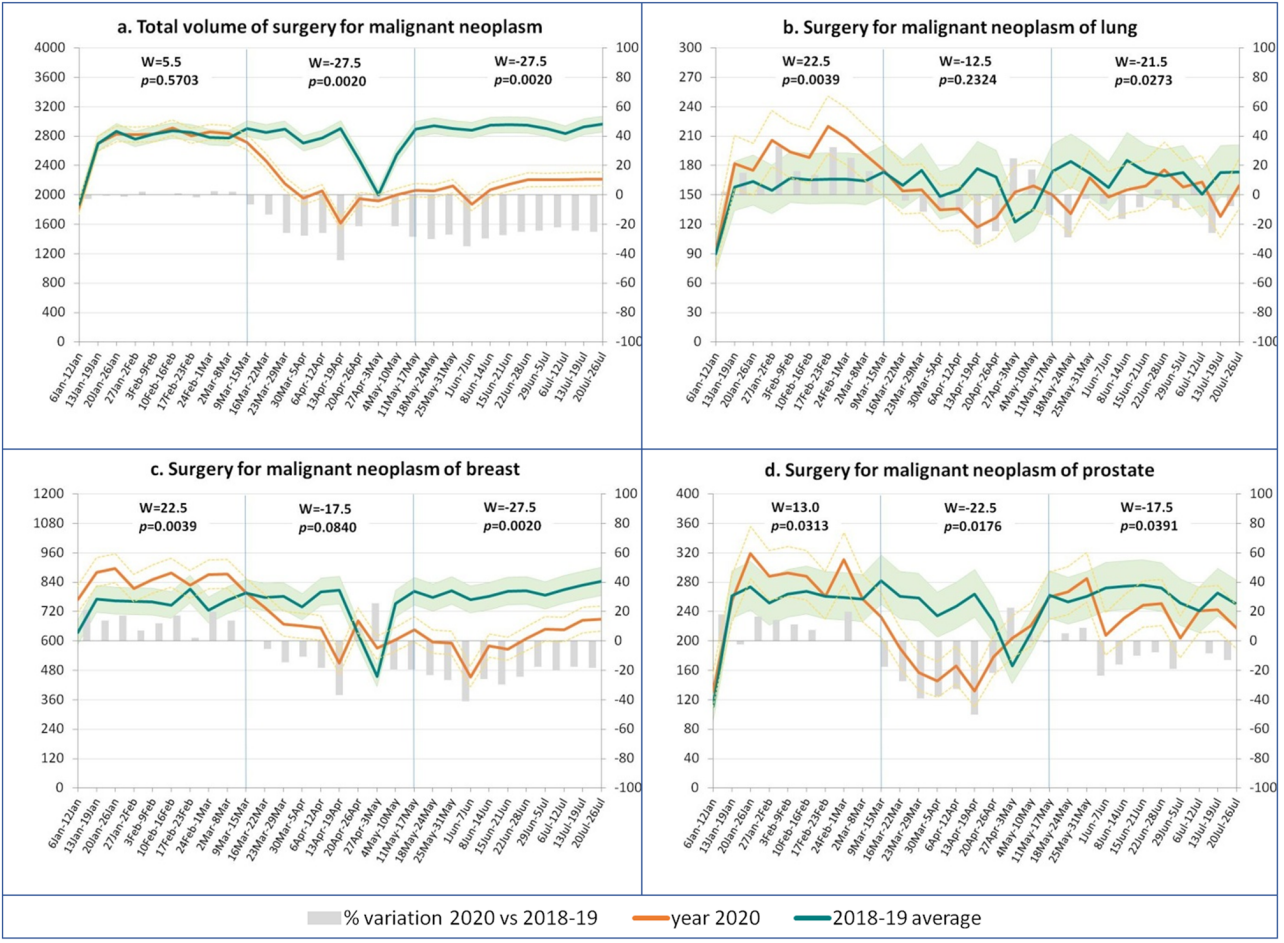
Impact of Covid-19 on oncological hospital care in Italy: surgery volumes by sub-period*. Weekly trend of indicators (left axis) with 95% CI and % variations (right axis)—January–July 2020 versus 2018–2019 average. *Pre-lockdown, lockdown (9/3–17/5), post-lockdown—W values and *p*-values from the paired-sample Wilcoxon test for comparisons within each sub-period.

The total volume of malignant cancers surgeries (Fig. 2a) showed a decline, which was moderate during the first 2 weeks of lockdown, reached − 25% by the end of March, and remained at these levels even in the post-lockdown. Trends differed by cancer site: the reduction in volumes was limited (less than 20%) for lung cancers (Fig. 2b), whereas it fell by about 30–40% for breast and prostate surgery (Fig. 2c, d), earlier for the latter and in the post-lockdown for the former.

### Orthopaedics

**Figure 3:**
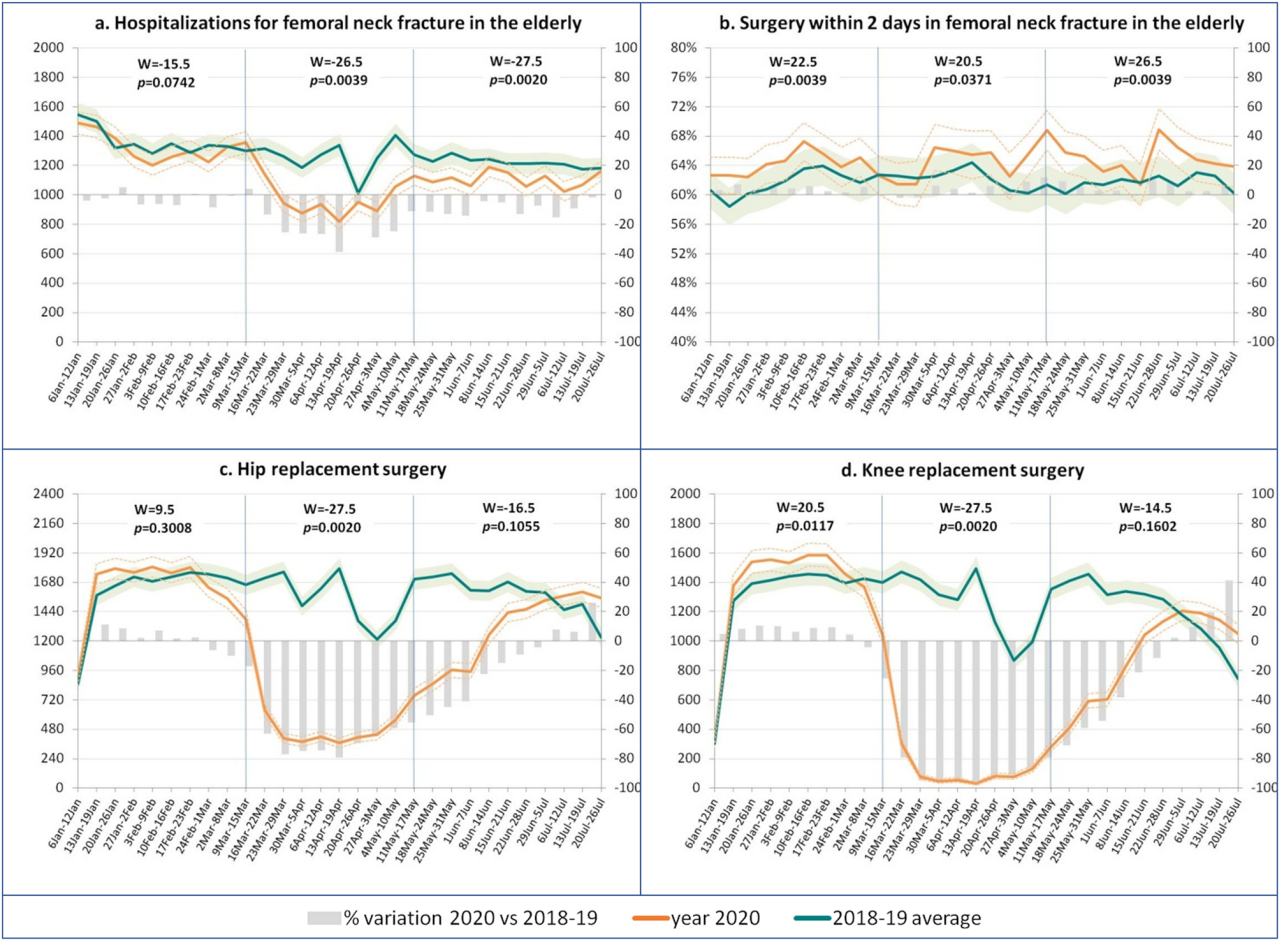
Impact of Covid-19 on orthopaedic hospital care in Italy: volumes and performance by sub-period*. Weekly trend of indicators (left axis) with 95% CI and % variations (right axis)—January–July 2020 versus 2018–2019 average. *Pre-lockdown, lockdown (9/3–17/5), post-lockdown—W values and *p*-values from the paired-sample Wilcoxon test for comparisons within each sub-period.

Hospitalizations for femoral neck fracture in the elderly (Fig. 3a) decreased by about 20% during lockdown, remaining significantly below the volumes of the previous 2 years in the post-lockdown too. In contrast, the percentage of timely interventions (Fig. 3b) was constantly higher than the average of the previous 2 years throughout the whole period, with the difference increasing in the post-lockdown. Hip and knee replacements plunged significantly during the lockdown period following national indications to suspend scheduled operations. In both cases, however, there was an inversion of the trends by the end of July 2020, with volumes significantly exceeding those recorded in 2018–2019.

## Discussion

In summary, hospitalizations have generally decreased for most diseases and surgical interventions, but the changes in hospital supply and organization have not jeopardized the capacity of a timely and effective response in time-dependent pathways of care throughout the period. However, volumes of both acute hospitalizations and elective surgery have not returned to the starting levels in the post-lockdown months, even for potentially severe conditions. General trends did not show important differences across regions, regardless of the different burden of Covid-19, with the exception of in-hospital mortality among STEMI patients in Lombardy.

The reduction in the hospitalizations for STEMI is likely to be the results of multiple intertwined factors. Along with the secular trend of reduced hospital access seen in the first months of 2020 and a potential lower incidence of heart attacks (e.g., related to better air quality or reduced mobility), delays in diagnosis or self-limitation of the demand by patients for fear of contagion may have played an important role. Indeed, the lack of timely access to hospital care would be supported by the reported increase in the number of out-of-hospital cardiac arrest deaths^5,6^. Moreover, we observed an increase in-hospital mortality among STEMI patients in Lombardy, where the high prevalence of Covid-19 was associated with worse STEMI outcomes^7^. This could result from a delay in seeking hospital care by patients suffering a heart attack, which, in turn, would undermine the efficacy of a primary PTCA.

The overall volumes of cancer surgery hide profound differences across sites: in each of them, in fact, the decision to postpone the surgery depends on the balance between the benefit of an immediate operation and the risk of hospital infection from Covid-19^8^. As expected, the decline in volumes of lung surgery was negligible, mainly due to its non-deferrable nature, while reductions for breast and prostate surgery were greater. Notably, especially for breast cancer, the persistence of the reduction in the post-lockdown is likely to be an indirect consequence of the suspension of screening activities^9^. The impact of such a contraction on women's health will only be visible in the future although recent simulation studies have already warned that delays in breast cancer surgery may be associated with an increased risk of death^10,11^.

Hospitalizations for femoral neck fracture in the elderly decreased, probably due to the limited mobility imposed by the confinement measures, while hip and knee replacements were heavily affected by the interruption of non-urgent surgery and the need to adapt surgical and outpatient activities to the new safety regulations^3^. This suspension has likely left room for an improvement in the timeliness management of emergencies, as in the case of the femoral neck fracture. Interestingly, the supply of orthopaedic surgery during the 2020 summer exceeded the volume of operations usually performed in this period. This reflects the timely and effective reorganisation of the sector after the lockdown in order to recover the accrued delays.

## Conclusion

Rescheduling less urgent interventions may not affect survival in the short term but may have amplified the severity of unresolved health problems. Furthermore, the deferral involves a lengthening of waiting lists that will require a rescheduling attentive to priorities and efficiency as well as equity, which is likely to be undermined by the direct and indirect effects of the pandemic. Moreover, our results suggest that reductions in hospital access were mainly driven by processes happening outside the hospital itself, even for severe conditions. It is therefore vital to strengthen preventive and primary care services so that they can adopt a pro-active approach and move towards the identification of at-risk conditions that were neglected during the pandemic and timely address patients to the secondary care system. 

Finally, the drop in cardiovascular or oncological care needs to be monitored more thoroughly to counteract a possible reduced access to early diagnosis (as in the case of breast cancer screening) and follow-up of severe chronic diseases.

## Supplementary Information

Supplementary Information 1.

Supplementary Figures.

## Data Availability

Individual anonymous data from health administrative databases have been processed at each regional health department, under appropriate privacy regulations, and only unidentifiable aggregated data have been shared with the team who carried out the pooled analysis. Authors followed the STROBE guidelines for reporting observational studies. The health administrative databases which are the data sources of this study are not publicly available; the unidentifiable aggregated data will be available from the corresponding author upon reasonable request.
