# Supplementary material for: Indirect impact of Covid-19 on hospital care pathways in Italy
Source: Sci Rep. 2021 Nov 2;11:21526. doi: 10.1038/s41598-021-00982-4 (PMC8563727; doi:10.1038/s41598-021-00982-4)
Supplement: Supplementary file 1 — Supplementary Information 1. [file 41598_2021_982_MOESM1_ESM.docx]

**Supplementary information 1 – List of indicators** **with their corresponding ICD-9-CM codes and selection criteria**

| **Figure** | **Indicator** | **International Classification of Diseases, Ninth Revision, Clinical Modification (ICD-9-CM Diseases) codes** |
| --- | --- | --- |
| Fig. 1a | Hospitalizations for STEMI | Diseases: 410.xx, excluding 410.7x, 410.9x |
| Fig. 1b | Hospitalizations for N-STEMI | Diseases: 410.7x, excluding 410.9x |
| Fig. 1c | PTCA interventions within 90’ in patients with STEMI | Diseases: 410.xx, excluding 410.7x, 410.9x and procedures within 90' from the admission: 00.66, 36.01, 36.02, 36.05, 36.06, 36.07 / hospitalizations for STEMI |
| Fig. 1d | In-hospital mortality in patients with STEMI | In-hospital deaths / hospitalizations for STEMI |
| Fig. 2a | Total volume of surgery for malignant neoplasm | **Esophagus** (diseases: 150.x, 151.0, 197.8 and procedures 42.4x), **Stomach** (diseases: 151.x, 197.8 and procedures: 43.5x-43.9x), **Colon** (diseases: 153.x, 197.5 and procedures: 45.7x, 45.8, 45.9x, 46.03, 46.04, 46.1x excluded diseases: 48.49, 48.5, 48.6), **Rectum** (diseases: 154.x, 197.5 and procedures: 48.49, 48.5, 48.6x excluded procedures: 45.7x, 45.8, 45.9x, 46.03, 46.04, 46.1x), **Liver** (diseases: 155.x, 197.7 and procedures: 50.22, 50.25, 50.29, 50.3, 50.4), **Gallbladder**: (diseases: 156.x, 197.8 and procedures: 51.22, 51.23), **Pancreas** (diseases: 157.x and procedures: 52.5x, 52.6, 52.7), **Lung** (diseases: 162.2, 162.3, 162.4, 162.5, 162.8, 162.9, 197.0 and procedures: 32.3, 32.4, 32.5, 32.6, 32.9, 32.29 ), **Bladder** (diseases: 188.x, 198.1 and procedures 57.6, 57.7x), **Kidney** (diseases: 189.x, 198.0 and procedures 55.4, 55.51, 55.52, 55.54), **Central nervous system** (diseases: 191.x, 192.x, 194.3, 194.4, 198.3, 198.4, 225.x, 227.3, 227.4, 237.0, 237.5, 239.6, 239.7 and procedures: 01.14, 01.23-01.25, 01.31, 01.39, 01.5x, 07.5x, 07.6x, 07.72), **Head and neck** (diseases: 140.x, 141.x,143.x-145.x, 141.0, 146.x, 148.x, 149.x, 161.x, 160.x, 147.x, 142.x, 196.0, 176.2, 195.0 and DRG 482), **Thyroid** (diseases: 193 and procedures: 06.2, 06.3x, 06.4, 06.5x, 06.6), **Breast** (diseases: 174.x, 198.81, 233.0 and procedures: 85.2x, 85.33, 85.34, 85.35, 85.36, 85.4x), **Uterus** (diseases: 179, 180.x, 182.x, 183.x, 184.x, 198.6, 198,.82 and procedures: 68.3x, 68.4x, 68.5x, 68.6x, 68.7x), **Prostate** (diseases: 185, 198.82 and procedures 60.3, 60.4, 60.5, 60.61, 60.62, 60.69 60.21, 60.29, 60.96, 60.97) |
| Fig. 2b | Surgery for malignant neoplasm of lung | Diseases: 162.2, 162.3, 162.4, 162.5, 162.8, 162.9, 197.0 and procedures: 32.3, 32.4, 32.5, 32.6, 32.9, 32.29 |
| Fig. 2c | Surgery for malignant neoplasm of breast | Diseases: 174.x, 198.81, 233.0 and procedures: 85.2x, 85.33, 85.34, 85.35, 85.36, 85.4x |
| Fig. 2d | Surgery for malignant neoplasm of prostate | Diseases: 185, 198.82 and procedures: 60.3, 60.4, 60.5, 60.61, 60.62, 60.69, 60.21, 60.29, 60.96, 60.97 |
| Fig. 3a | Hospitalizations for femoral neck fracture in the elderly | Diseases: 820.xx and age >=65 (excluding rehabilitation and long-term care) |
| Fig. 3b | Surgery within 2 days in femoral neck fracture in the elderly | Diseases: 820.xx and procedures within 2 days from the admission: 81.51, 81.52, 79.00, 79.05, 79.10, 79.15, 79.20, 79.25, 79.30, 79.35, 79.40, 79.45, 79.50, 79.55 / hospitalizations for femoral neck fracture in the elderly |
| Fig. 3c | Hip replacement surgery | Procedures: 81.51, 81.52, 81.53, 00.70, 00.71, 00.72, 00.73, 00.85, 00.86, 00.87 |
| Fig. 3d | Knee replacement surgery | Procedures: 81.54, 81.55, 00.80, 00.81, 00.82, 00.83, 00.84 |
