# Supplementary material for: Indirect impact of Covid-19 on hospital care pathways in Italy
Source: Sci Rep. 2021 Nov 2;11:21526. doi: 10.1038/s41598-021-00982-4 (PMC8563727; doi:10.1038/s41598-021-00982-4)
Supplement: Supplementary file 2 — Supplementary Figures. [file 41598_2021_982_MOESM2_ESM.docx]

**Supplementary information 2 – Figures S1-S3 with detailed regional indicators**

**Figure S1a. Hospitalizations for STEMI: volumes by region and sub-period***

Weekly trend of indicators (left axis) and percent variations (right axis) – Jan-Jul 2020 vs. 2018-19 average

| 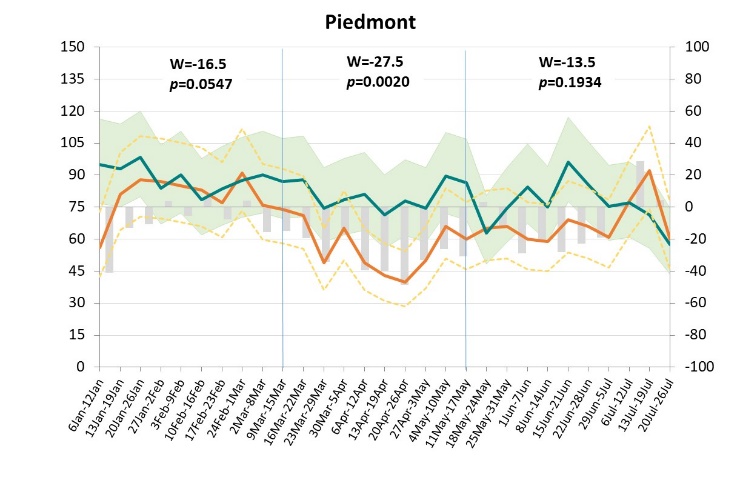 | 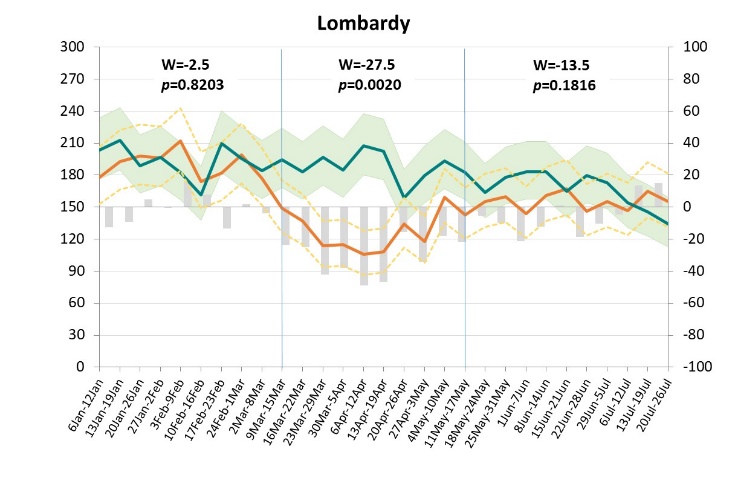 |
| --- | --- |
| 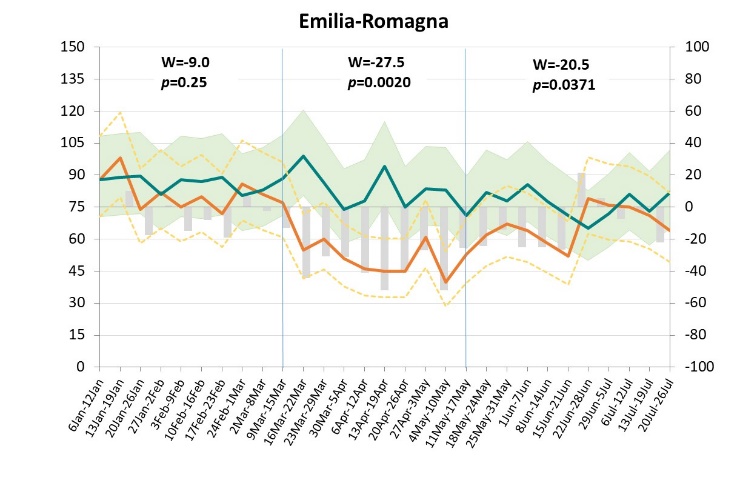 | 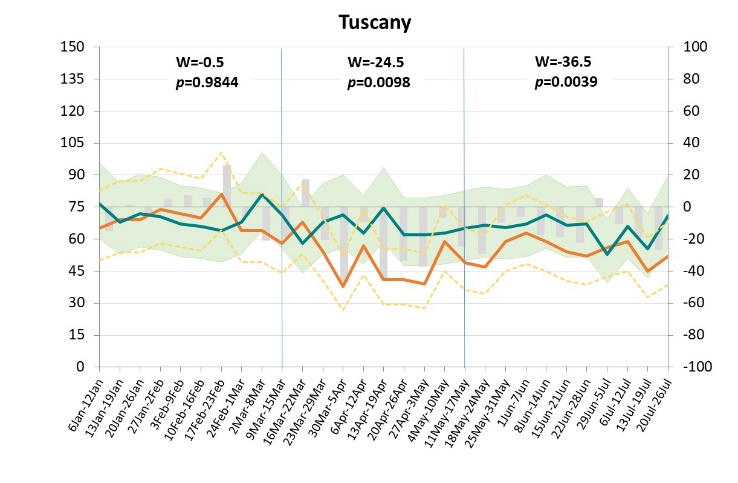 |
| 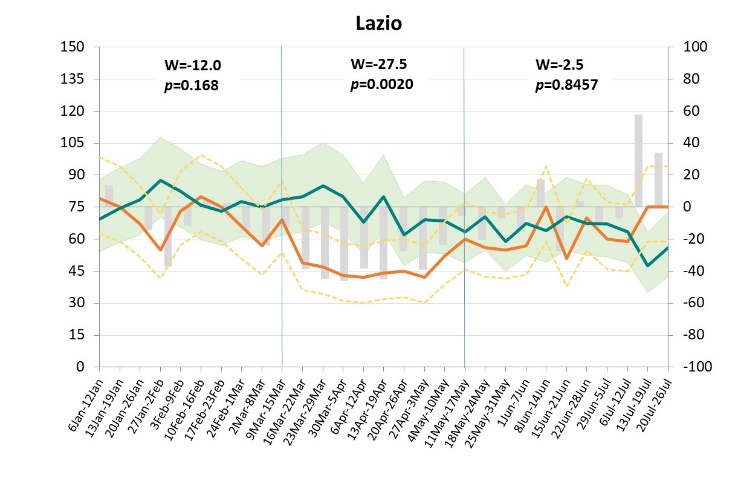 | 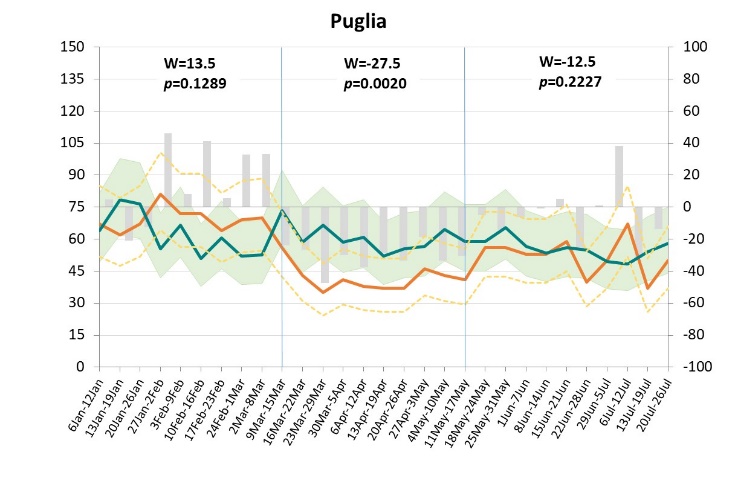 |
| 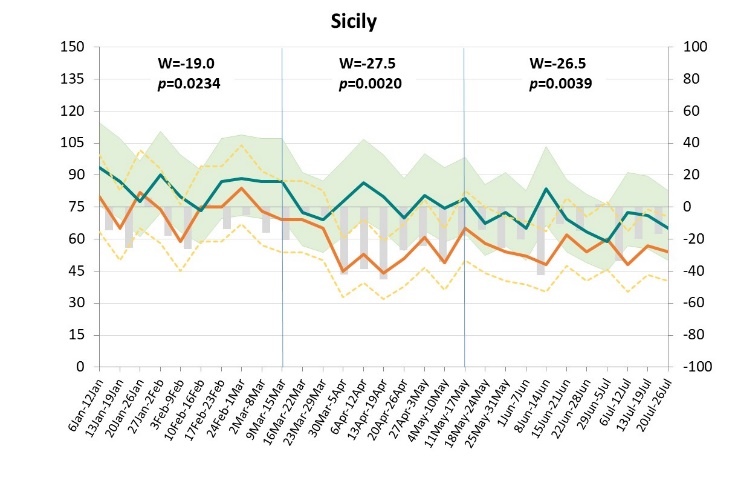 | |
| 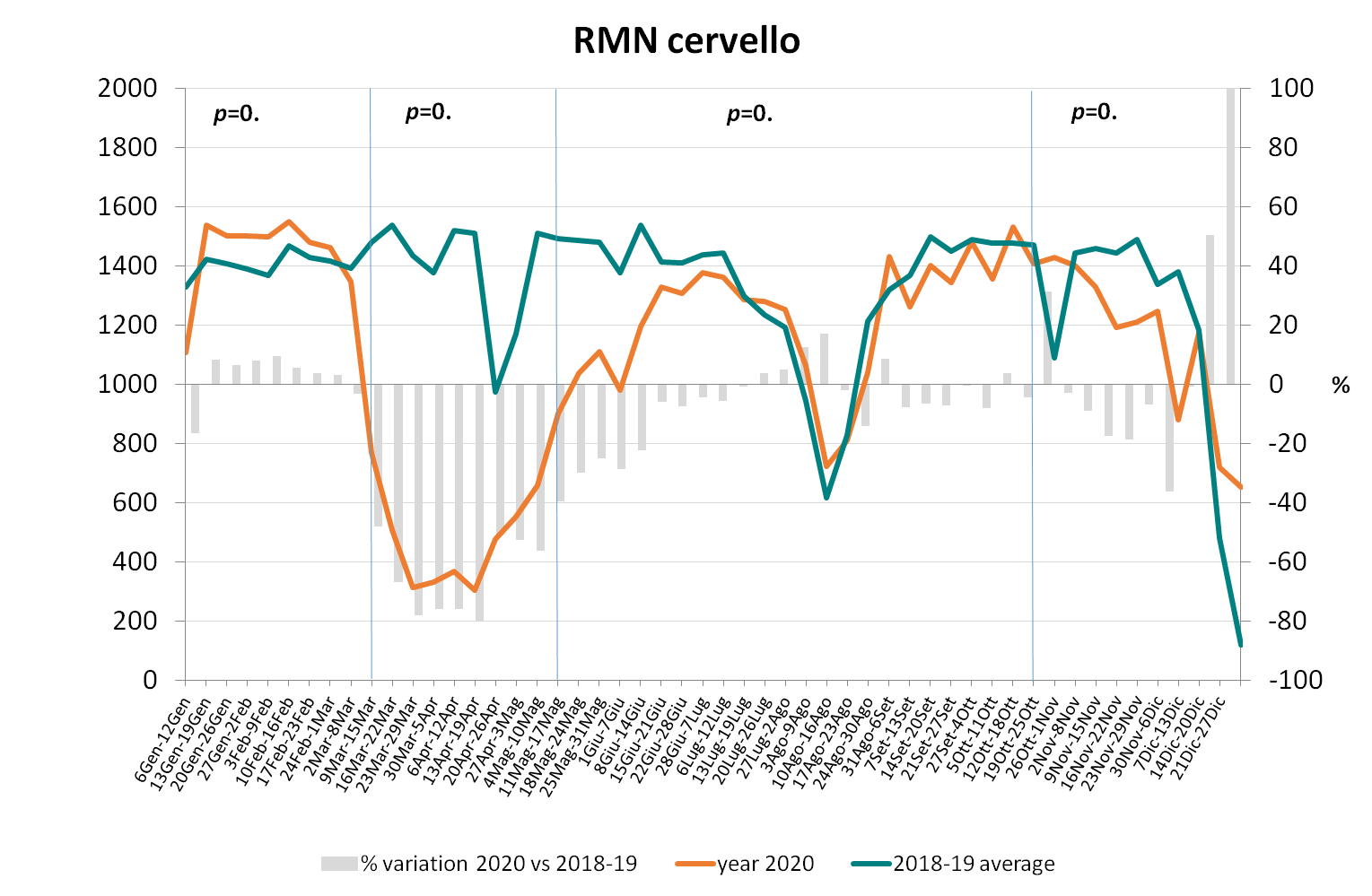 | |

* pre-lockdown, lockdown (9/3-17/5), post-lockdown – W values and p-values from the paired-sample Wilcoxon test for comparisons within each sub-period

**Figure S1b. Hospitalizations for N-STEMI: volumes by region and sub-period***

Weekly trend of indicators (left axis) and percent variations (right axis) – Jan-Jul 2020 vs. 2018-19 average

| 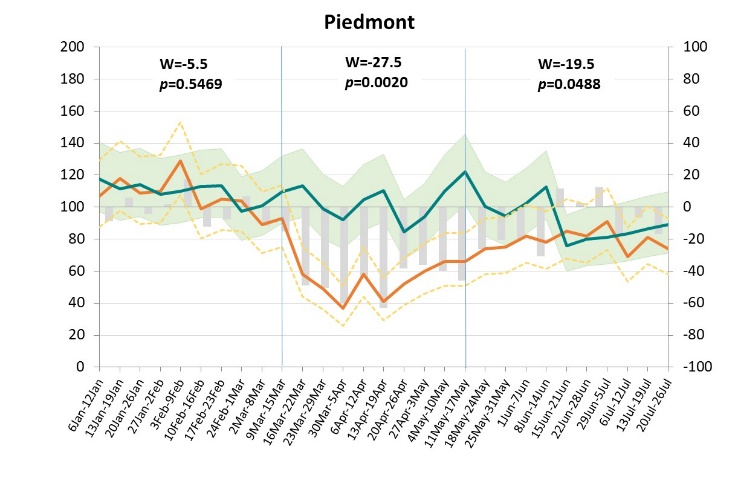 | 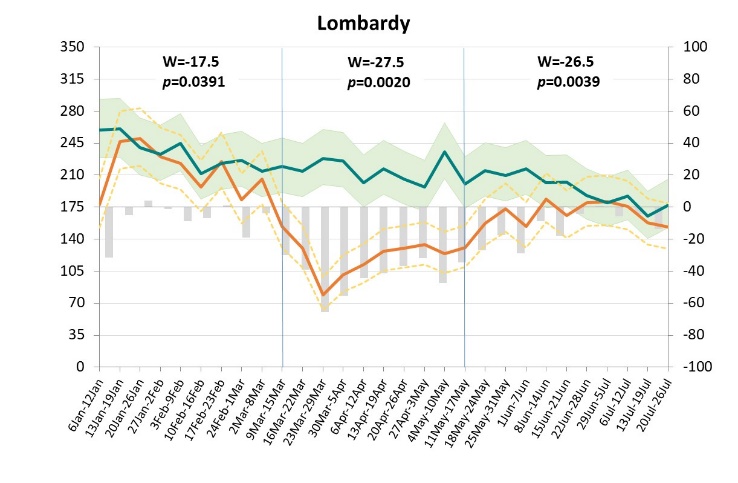 |
| --- | --- |
| 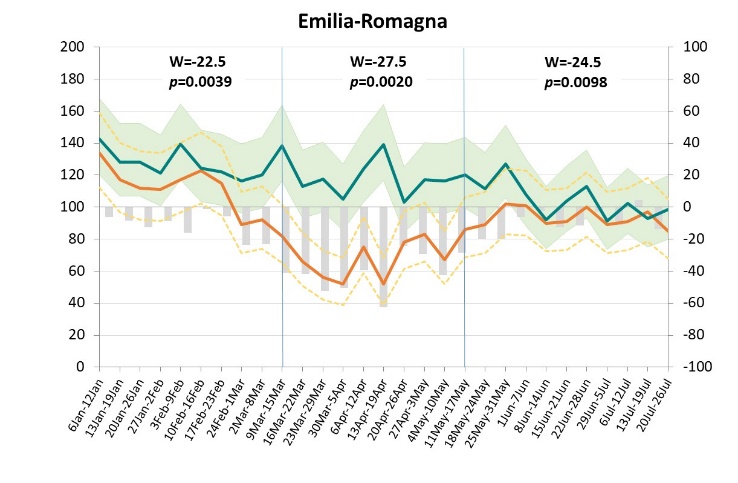 | 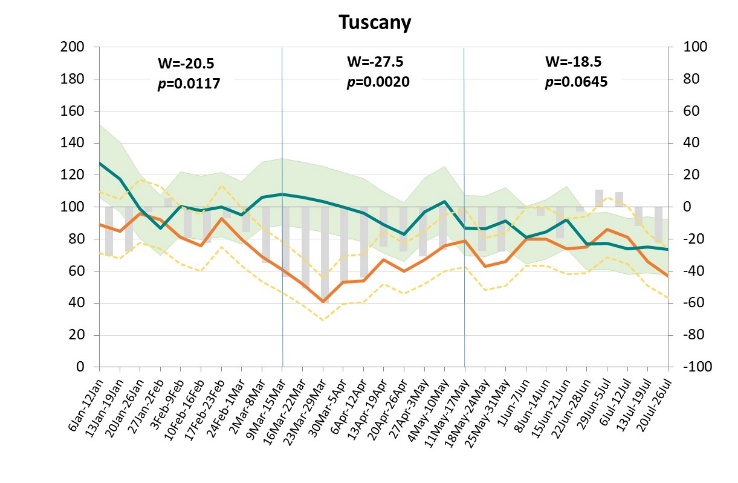 |
| 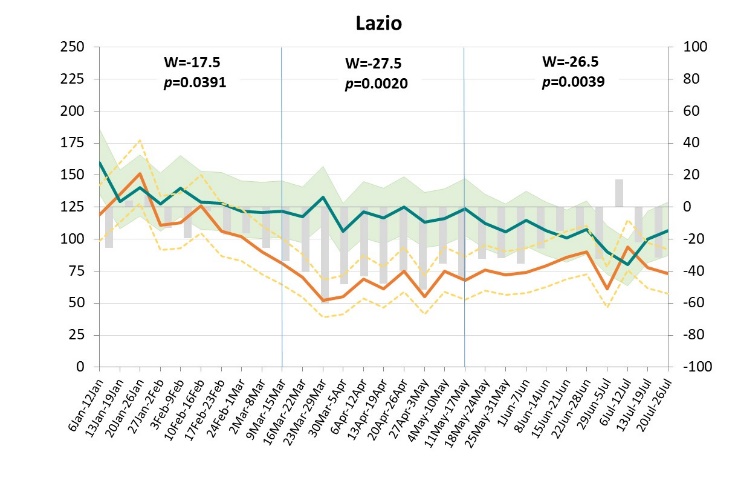 | 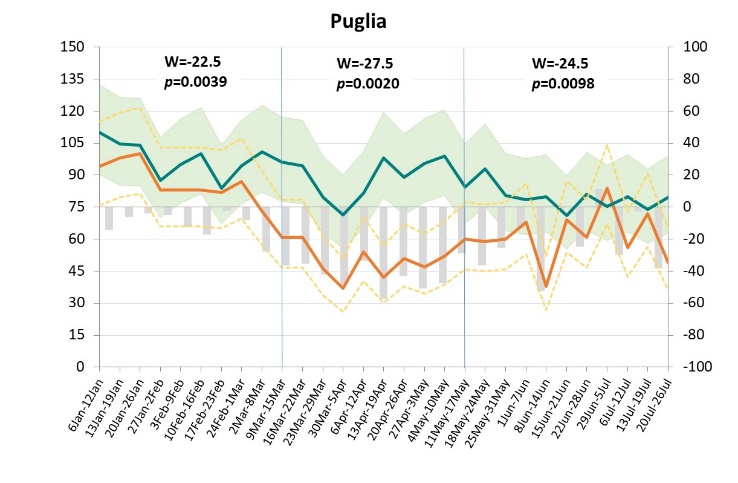 |
| 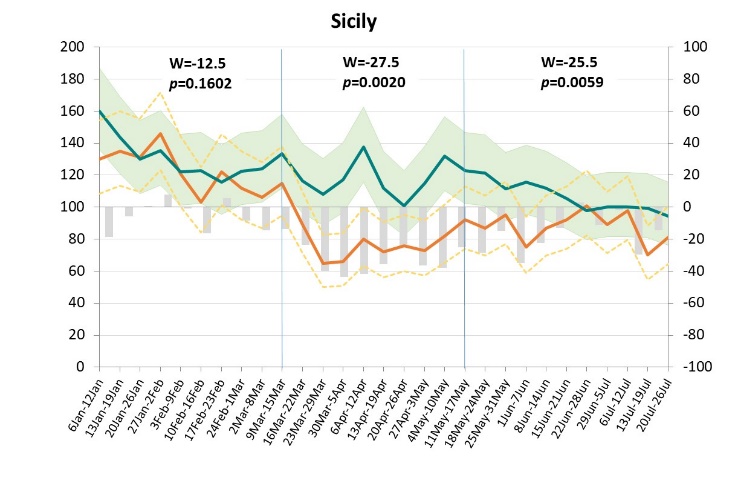 | |
| 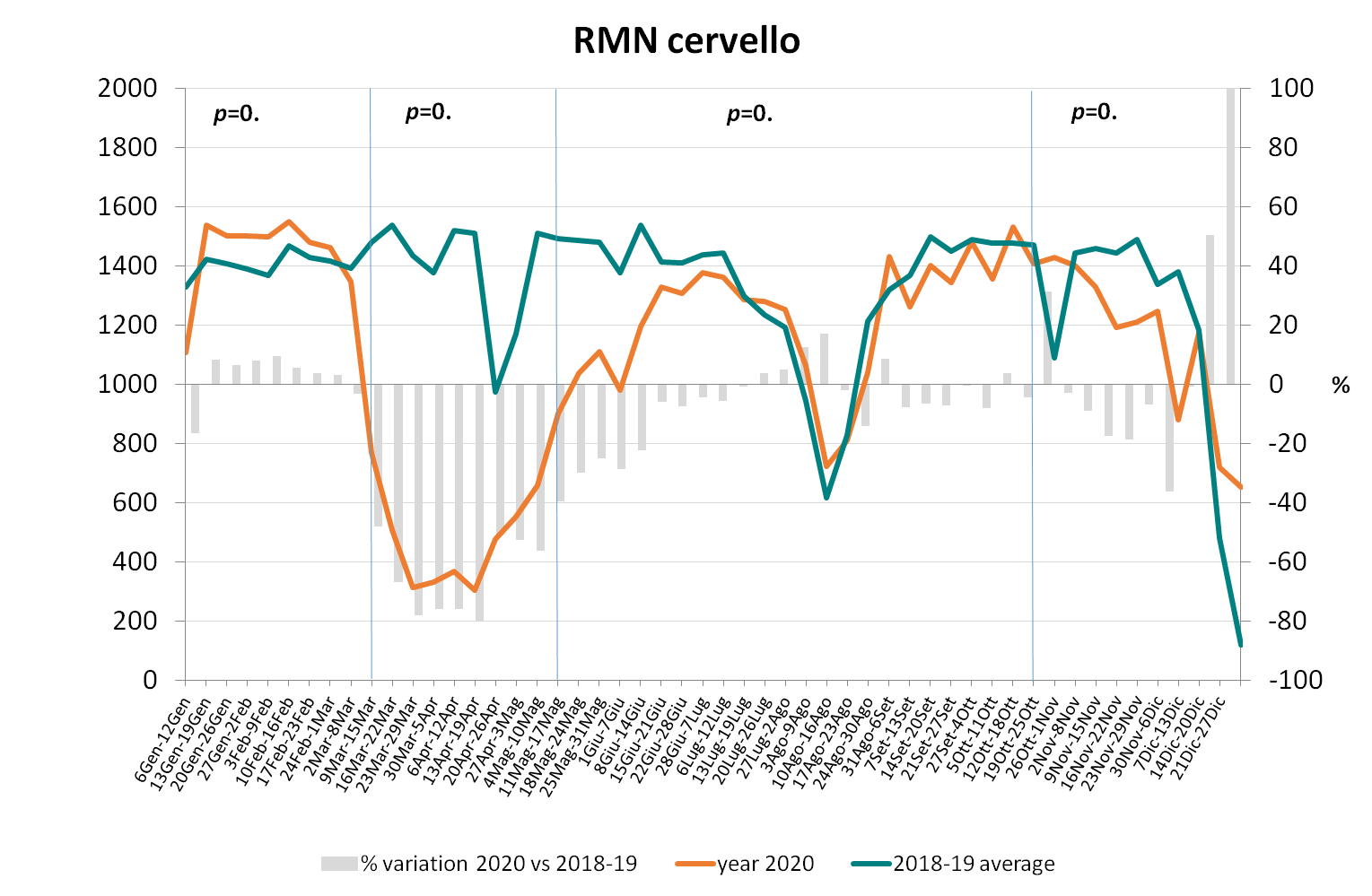 | |

* pre-lockdown, lockdown (9/3-17/5), post-lockdown – W values and p-values from the paired-sample Wilcoxon test for comparisons within each sub-period

**Figure S1c. PTCA interventions within 90’ in patients with STEMI: performance by region and sub-period***

Weekly trend of indicators (left axis) and percent variations (right axis) – Jan-Jul 2020 vs. 2018-19 average

| 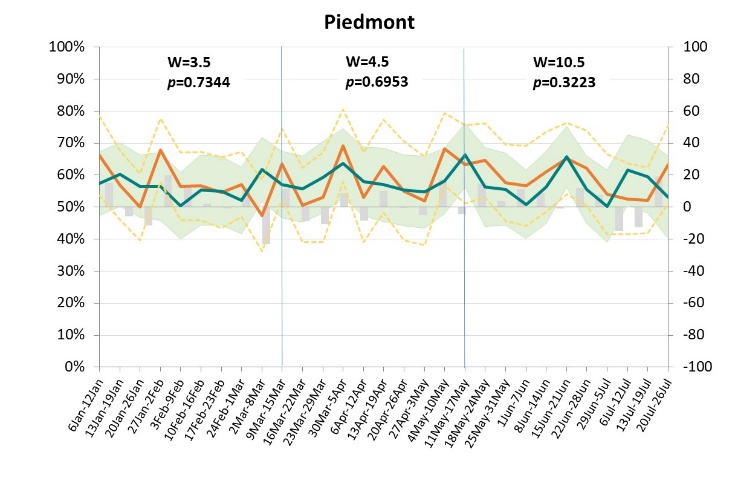 | 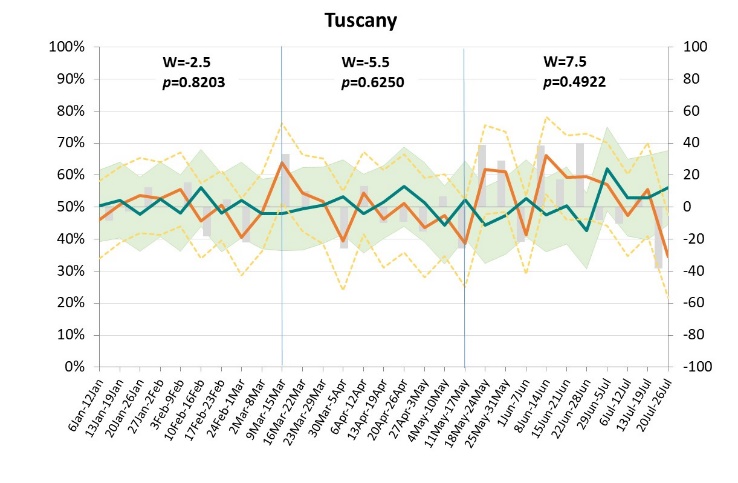 |
| --- | --- |
| 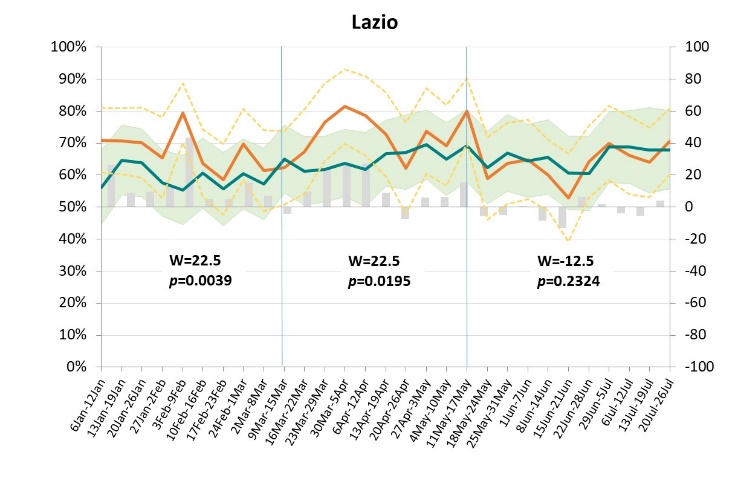 | 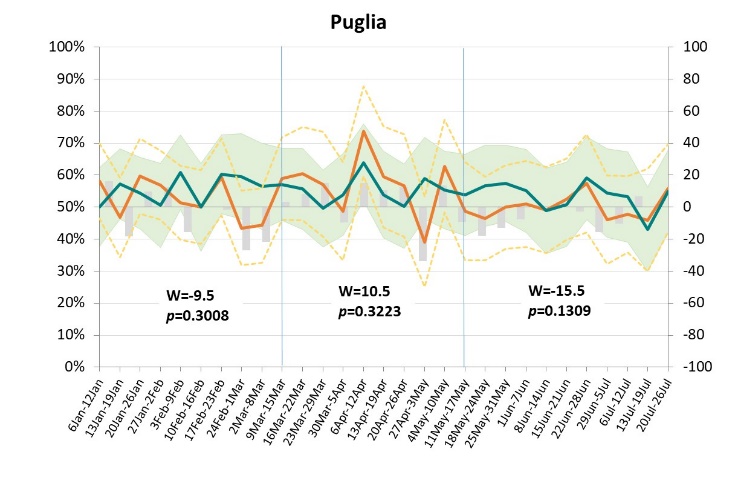 |
| 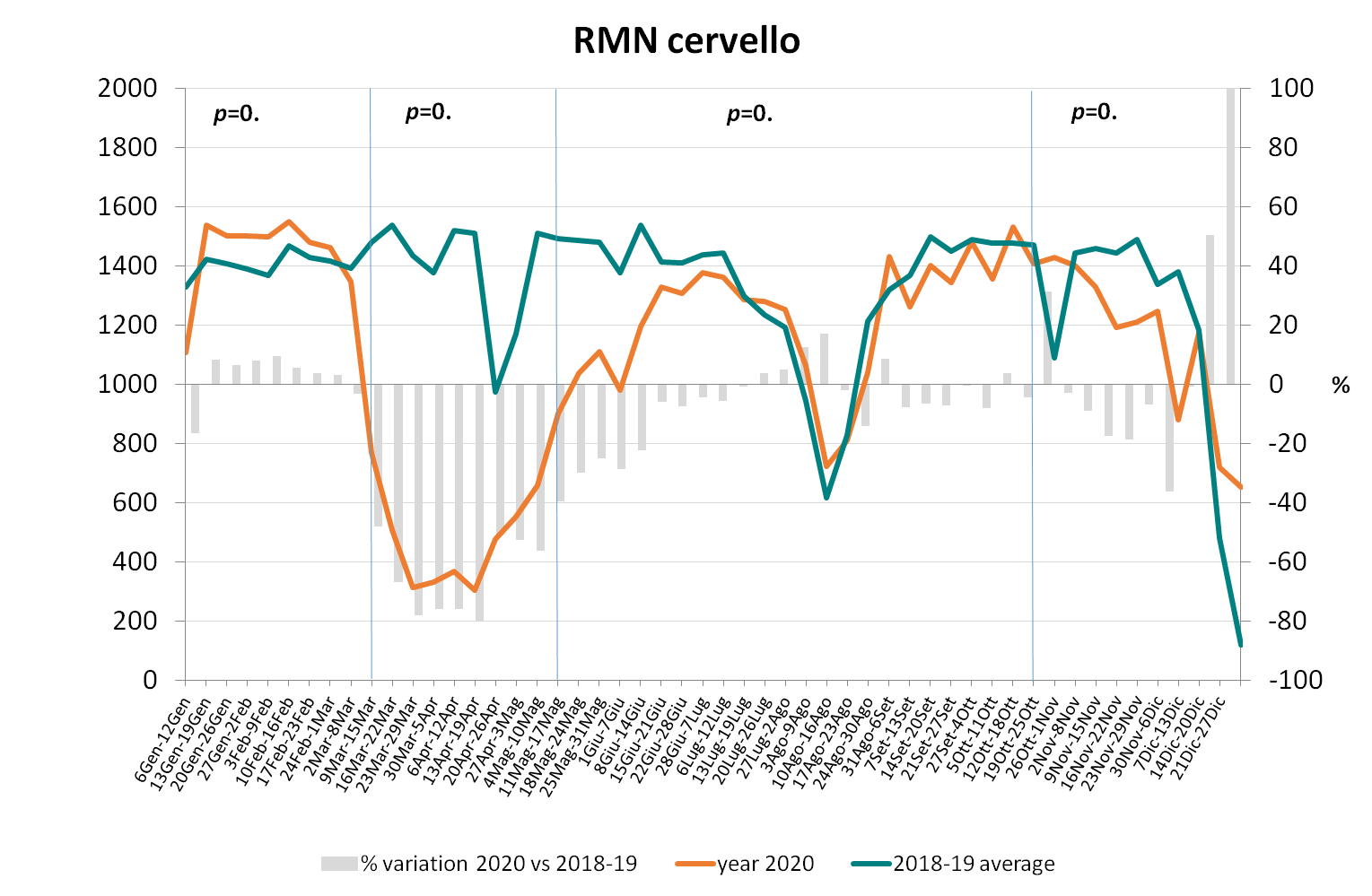 | |

* pre-lockdown, lockdown (9/3-17/5), post-lockdown – W values and p-values from the paired-sample Wilcoxon test for comparisons within each sub-period

**Figure S1d. In-hospital mortality in patients with STEMI: performance by region and sub-period***

Weekly trend of indicators (left axis) and percent variations (right axis) – Jan-Jul 2020 vs. 2018-19 average

| 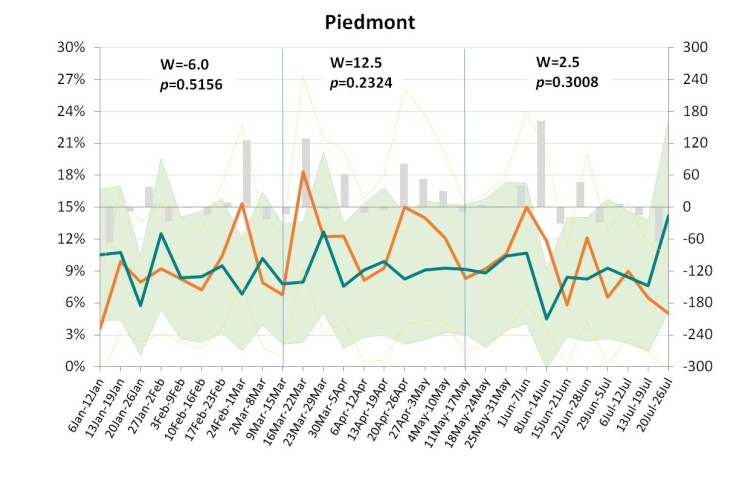 | 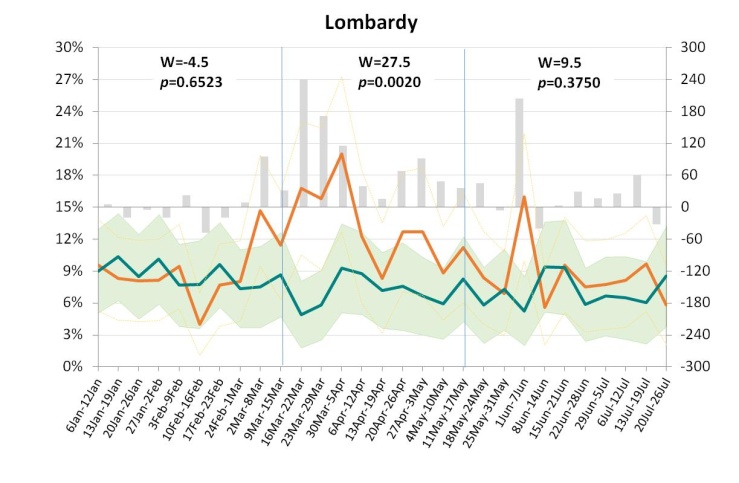 |
| --- | --- |
| 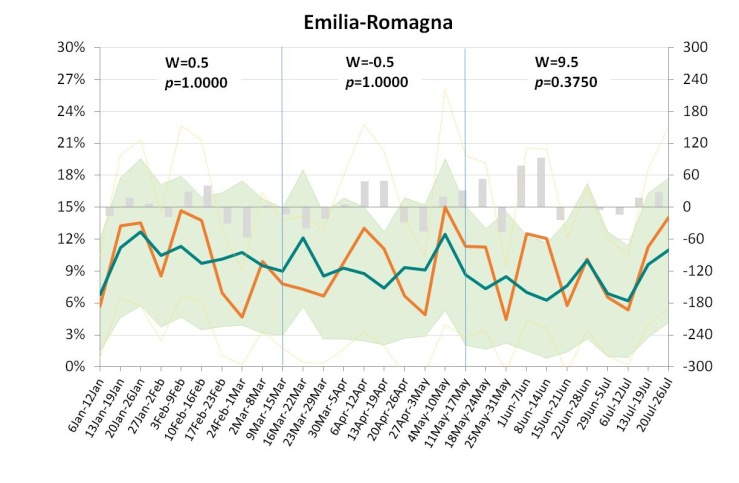 | 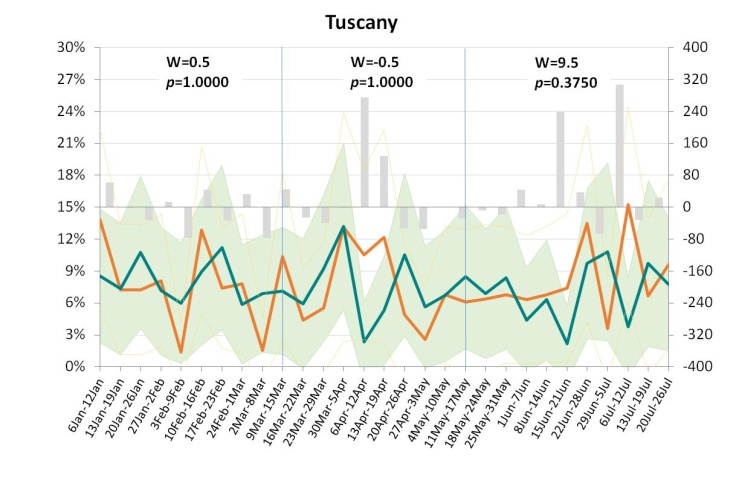 |
| 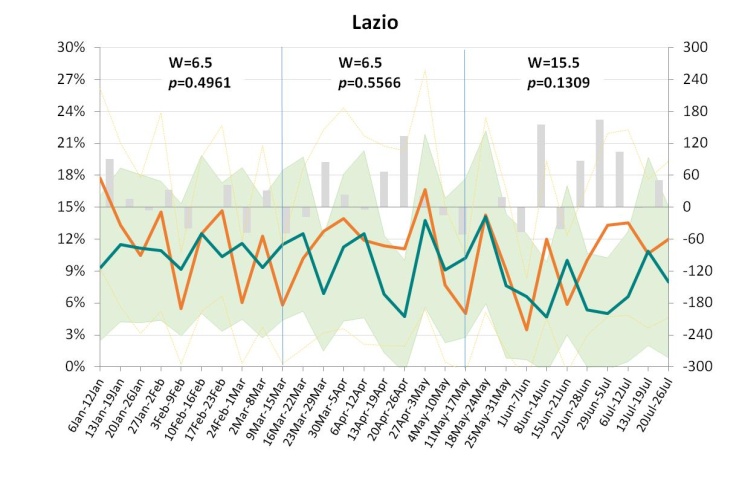 | 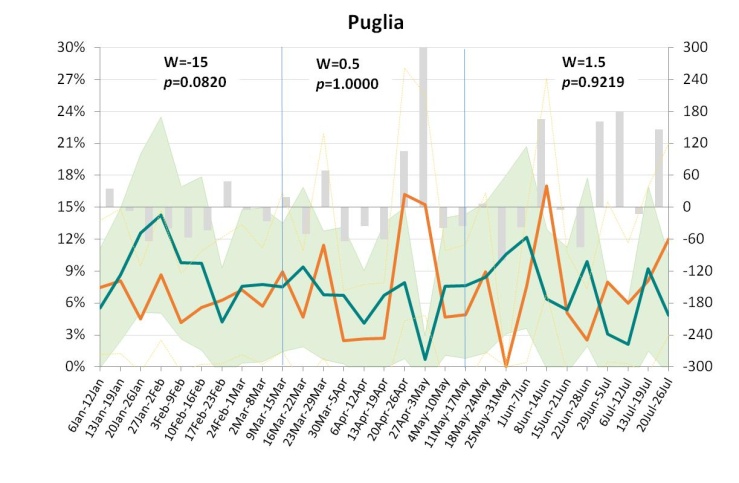 |
| 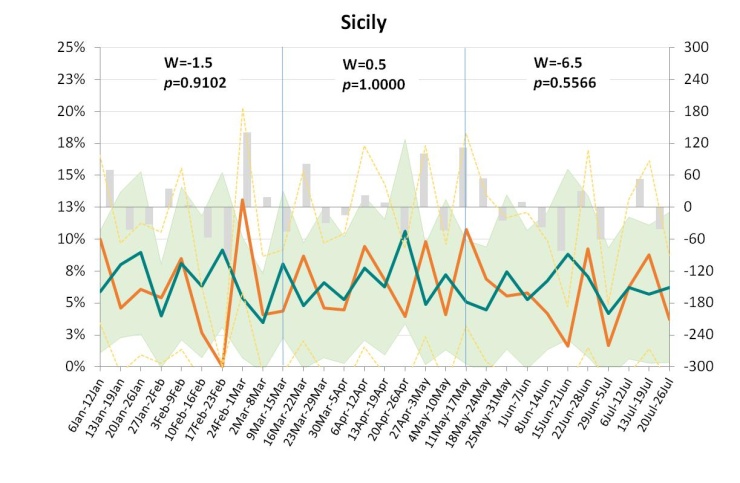 | |
| 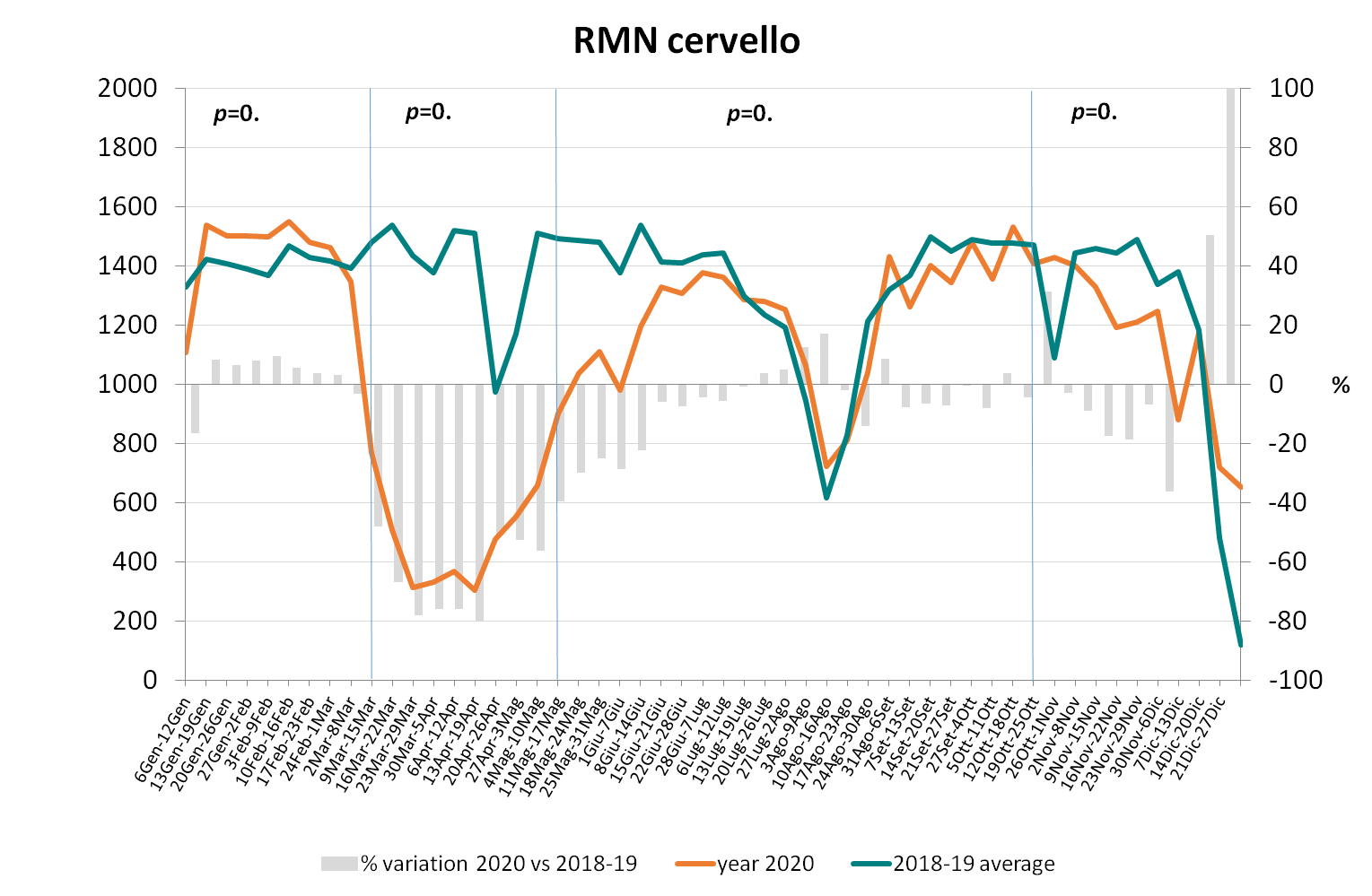 | |

* pre-lockdown, lockdown (9/3-17/5), post-lockdown – W values and p-values from the paired-sample Wilcoxon test for comparisons within each sub-period

**Figure S2a. Total volume of surgery for malignant neoplasm: volumes by region and sub-period***

Weekly trend of indicators (left axis) and percent variations (right axis) – Jan-Jul 2020 vs. 2018-19 average

| 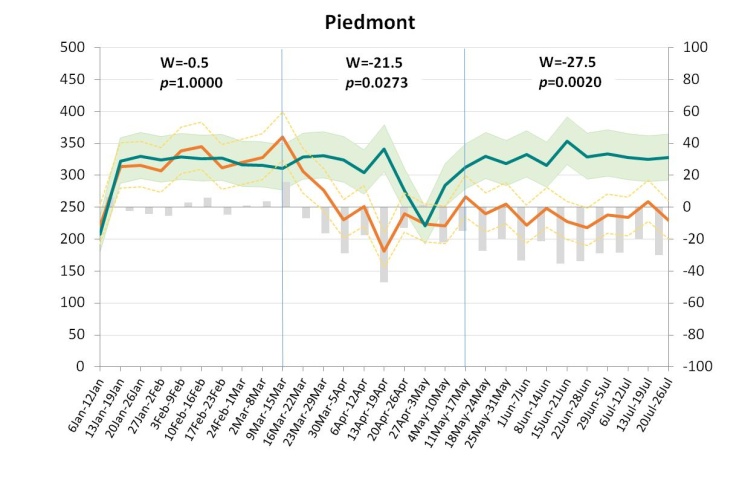 | 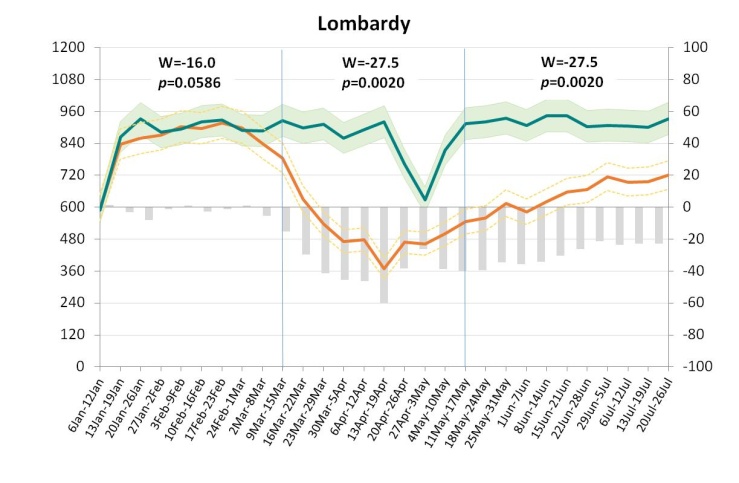 |
| --- | --- |
| 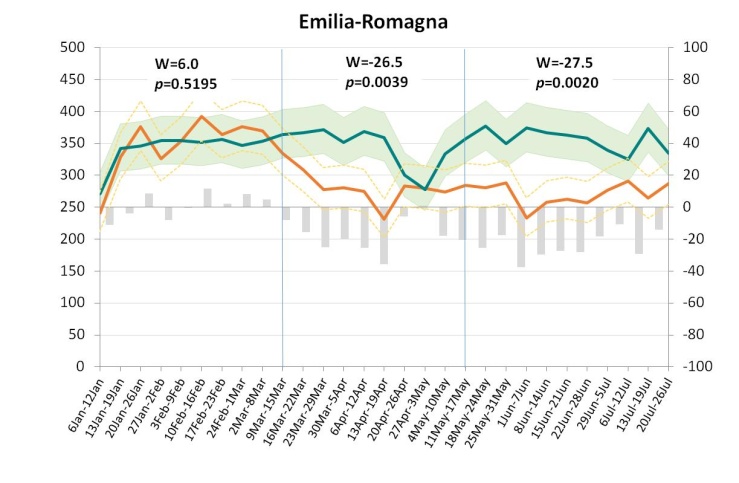 | 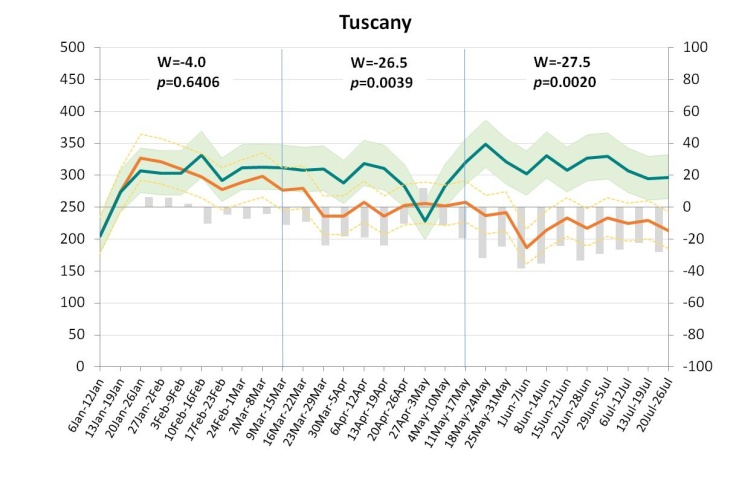 |
| 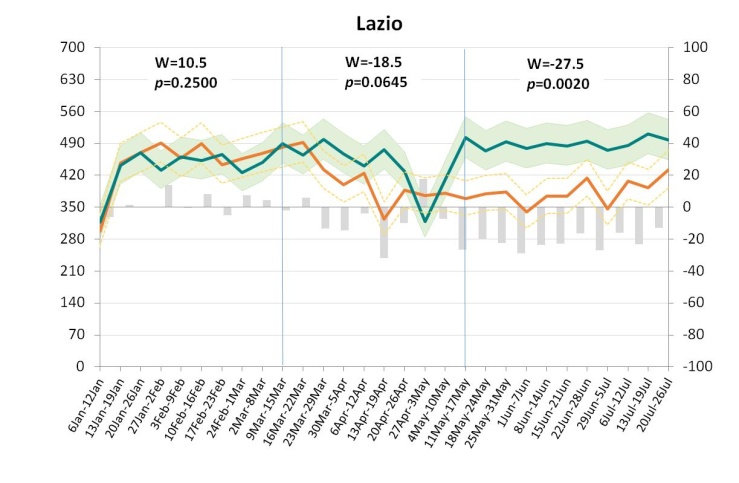 | 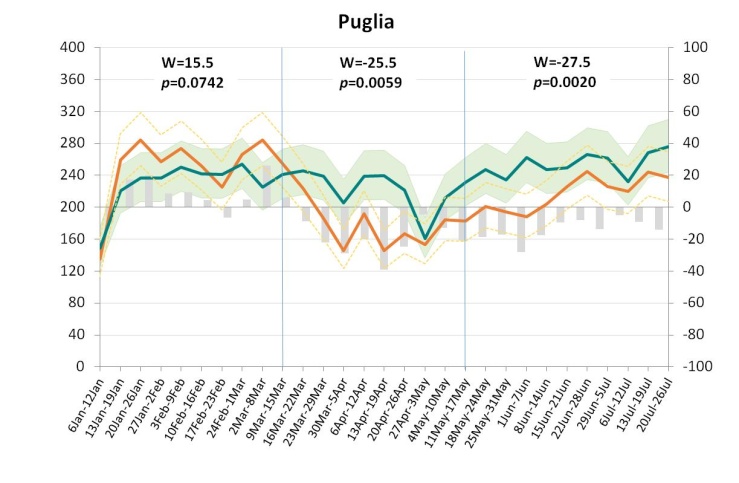 |
| 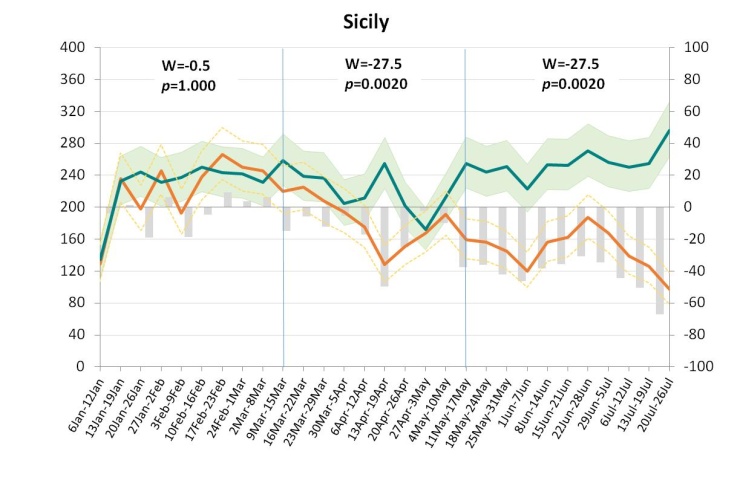 | |
| 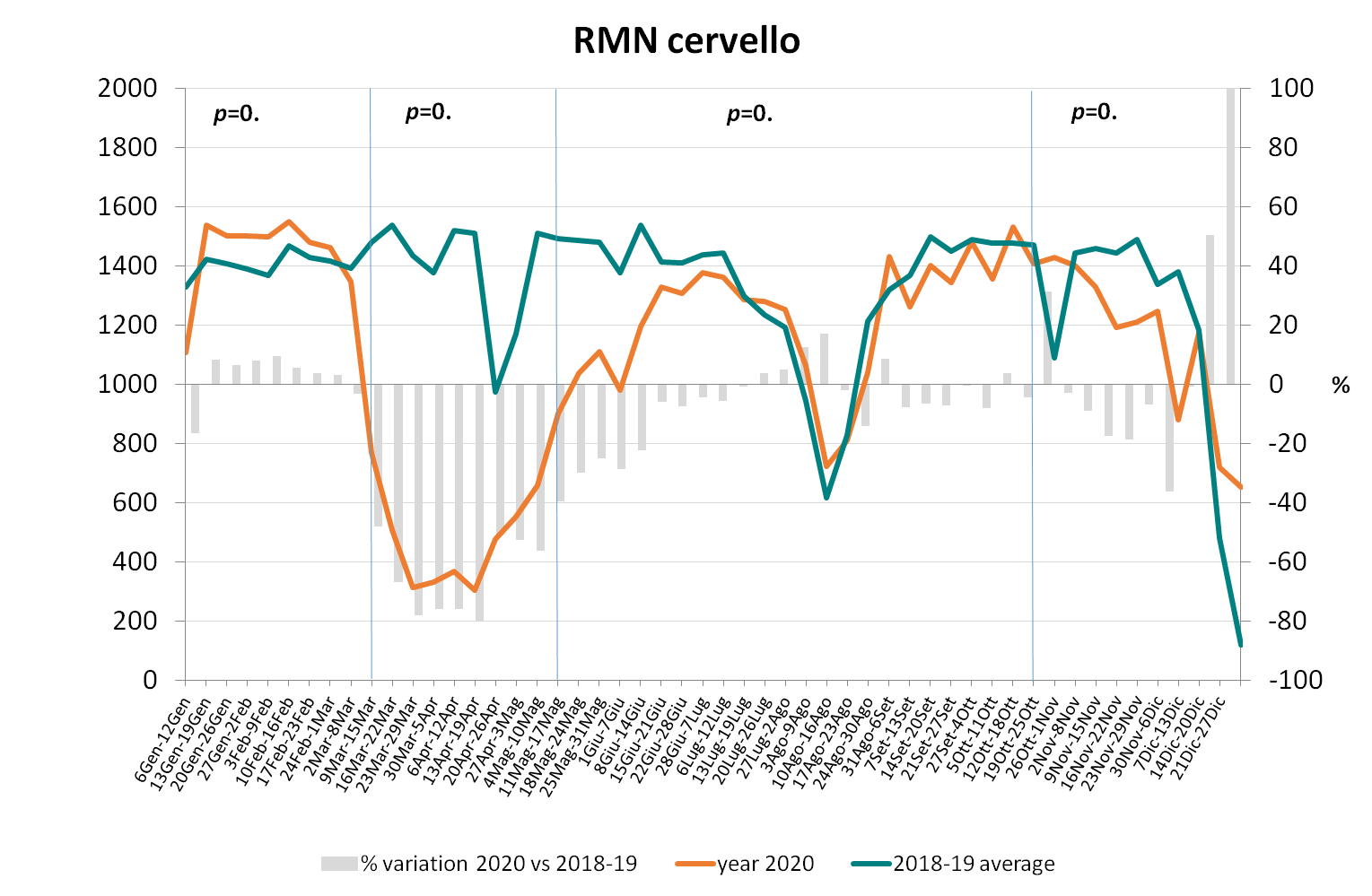 | |

* pre-lockdown, lockdown (9/3-17/5), post-lockdown – W values and p-values from the paired-sample Wilcoxon test for comparisons within each sub-period

**Figure S2b. Surgery for malignant neoplasm of lung: volumes by region and sub-period***

Weekly trend of indicators (left axis) and percent variations (right axis) – Jan-Jul 2020 vs. 2018-19 average

| 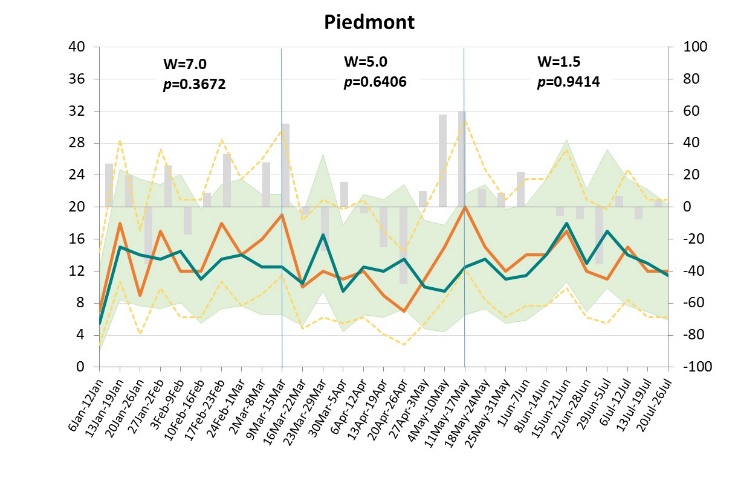 | 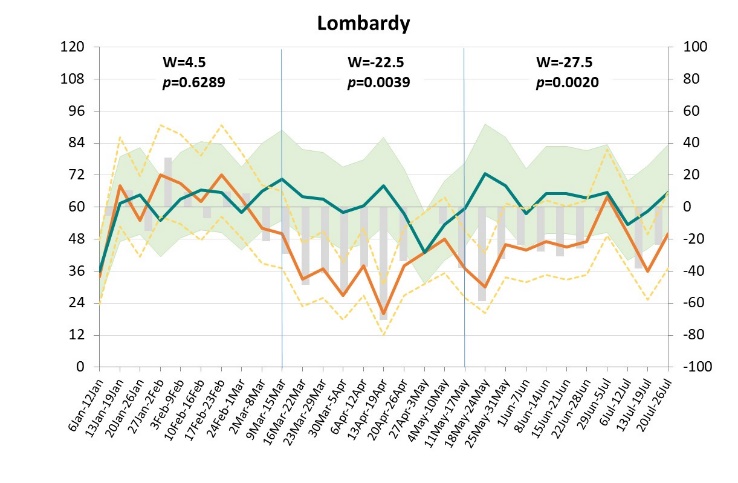 |
| --- | --- |
| 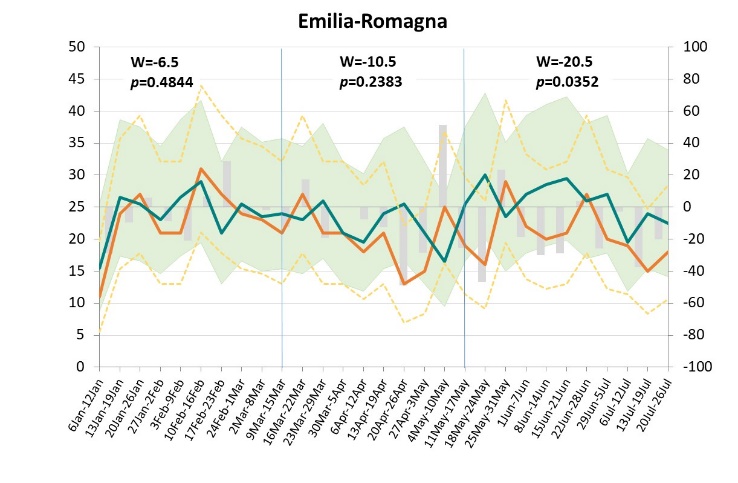 | 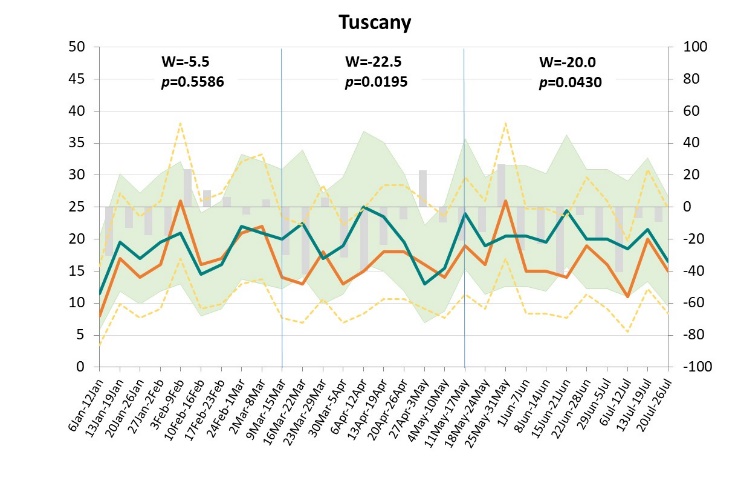 |
| 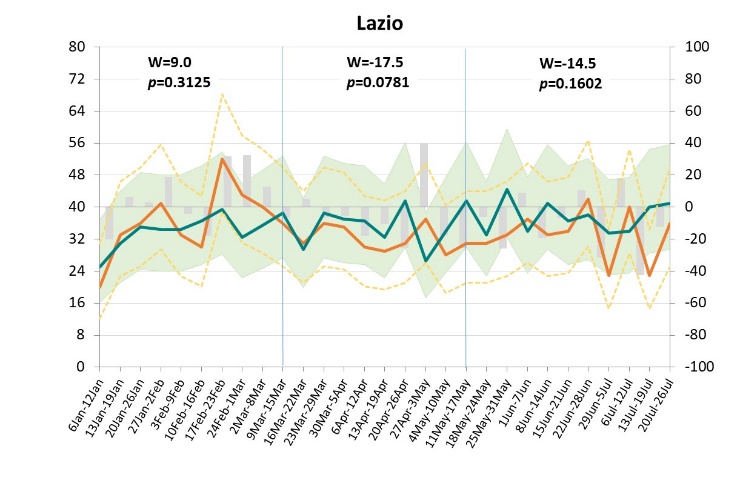 | 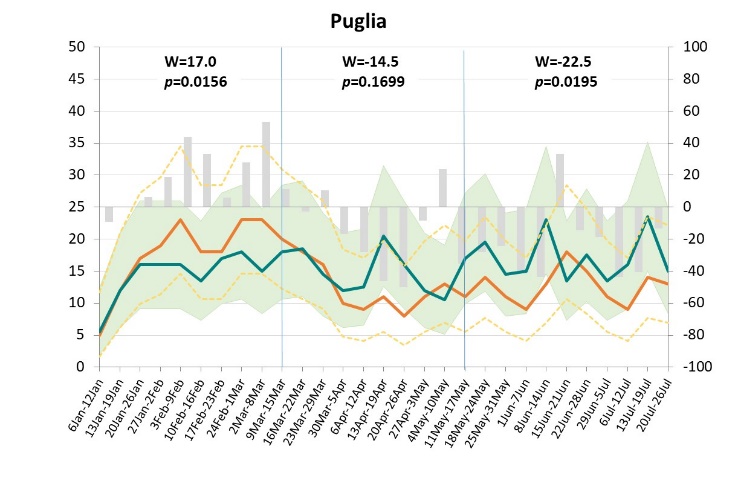 |
| 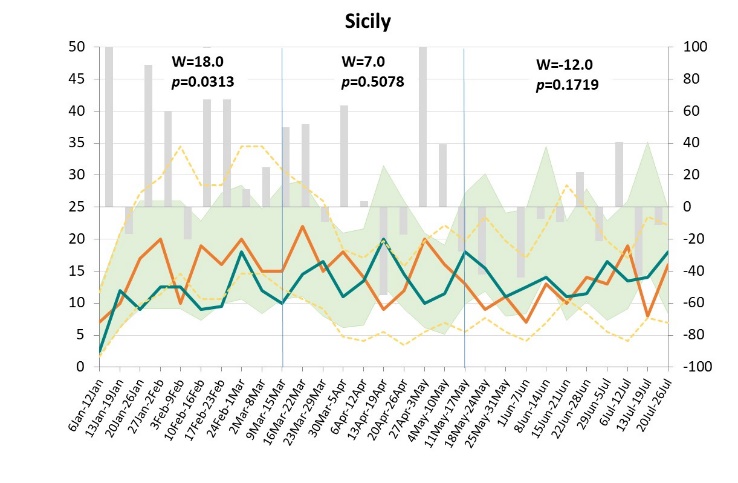 | |
| 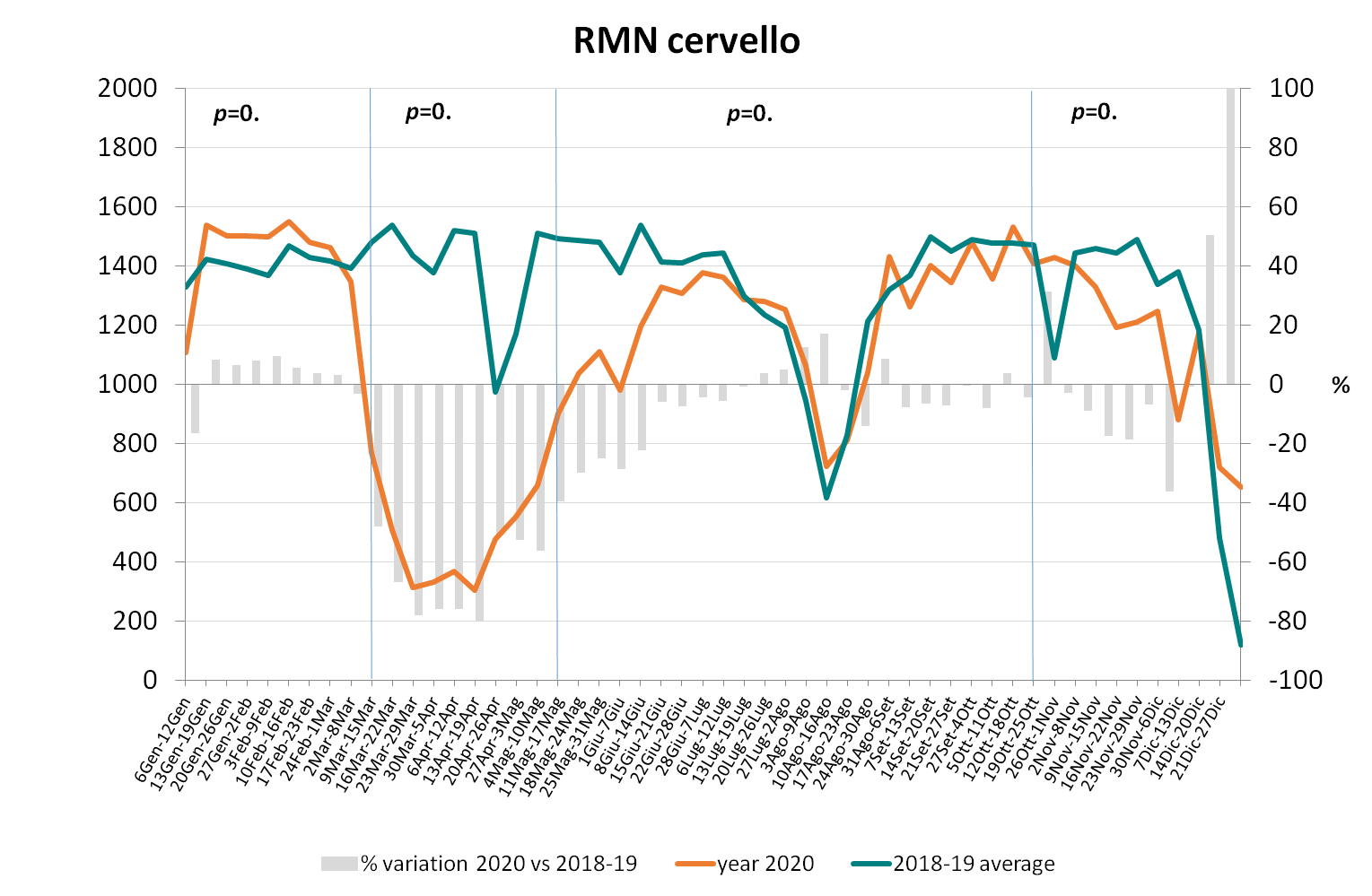 | |

* pre-lockdown, lockdown (9/3-17/5), post-lockdown – W values and p-values from the paired-sample Wilcoxon test for comparisons within each sub-period

**Figure S2c. Surgery for malignant neoplasm of breast: volumes by region and sub-period***

Weekly trend of indicators (left axis) and percent variations (right axis) – Jan-Jul 2020 vs. 2018-19 average

| 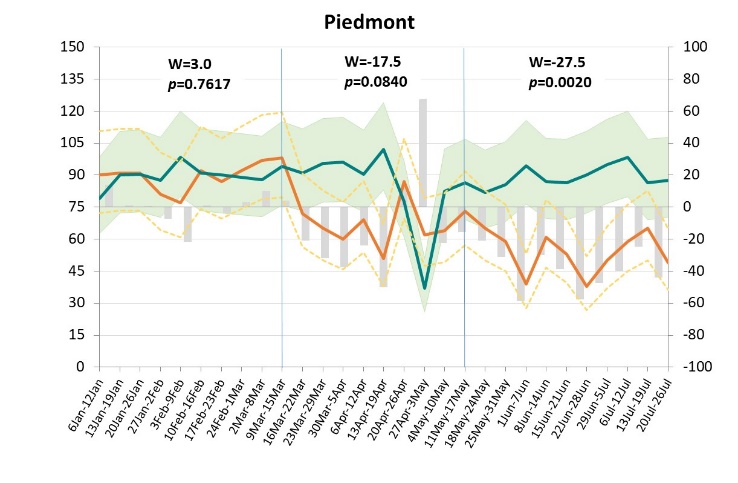 | 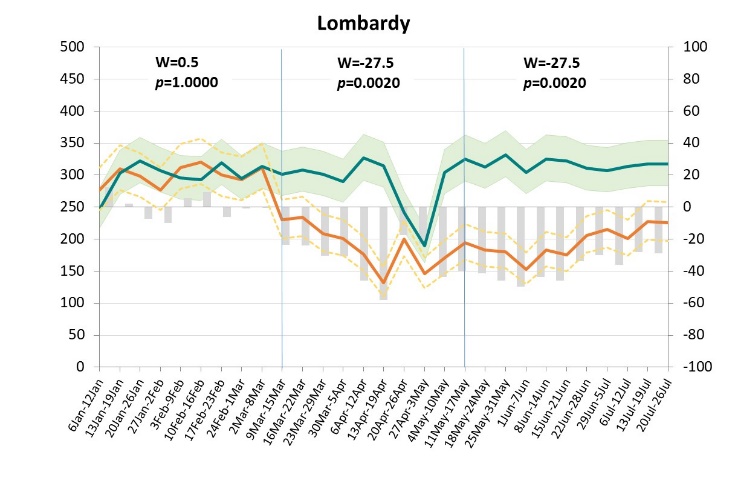 |
| --- | --- |
| 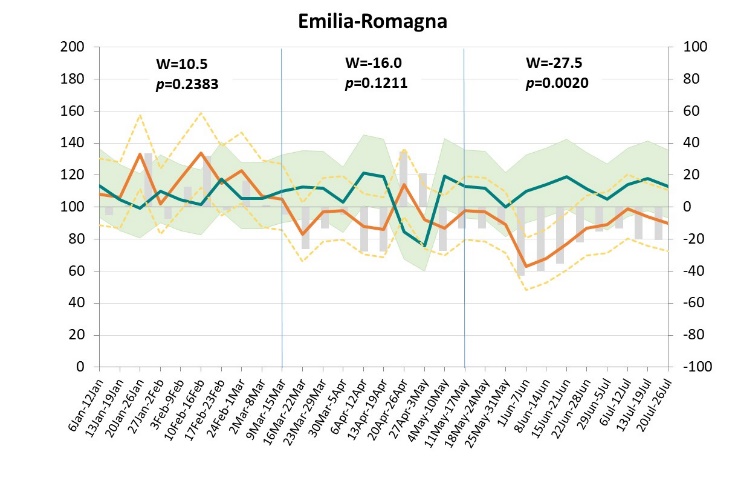 | 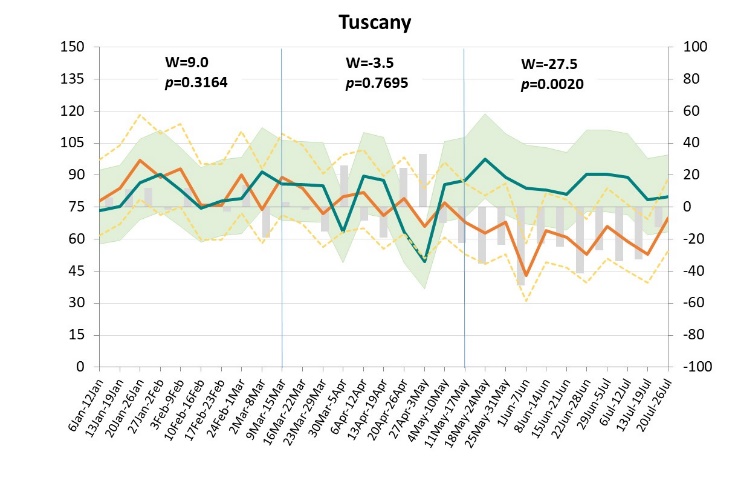 |
| 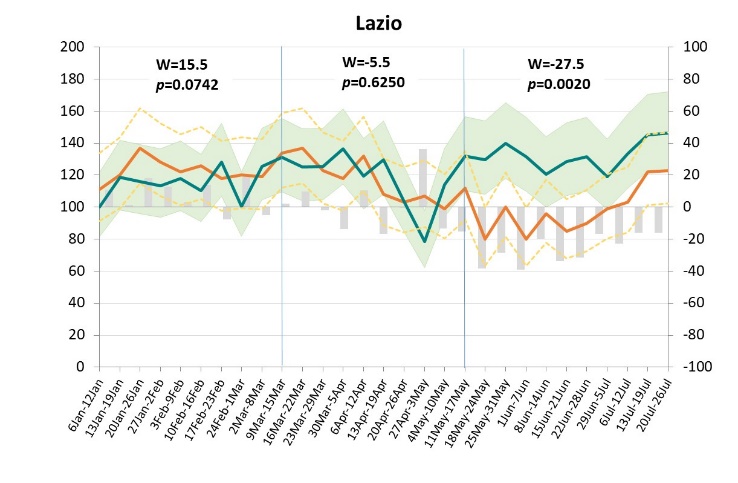 | 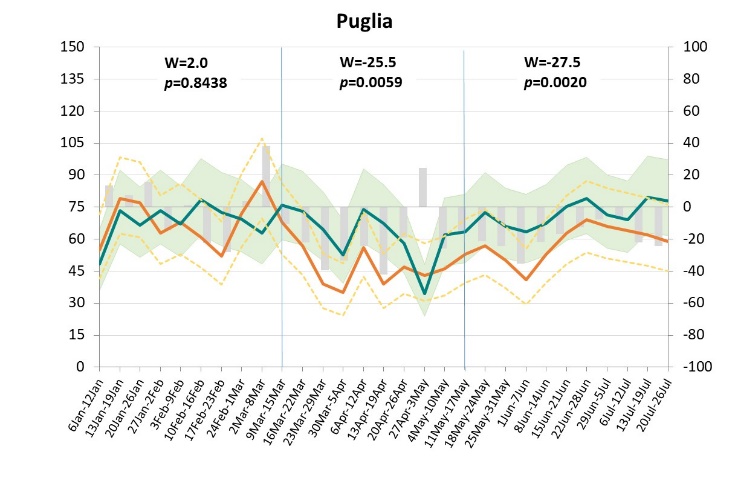 |
| 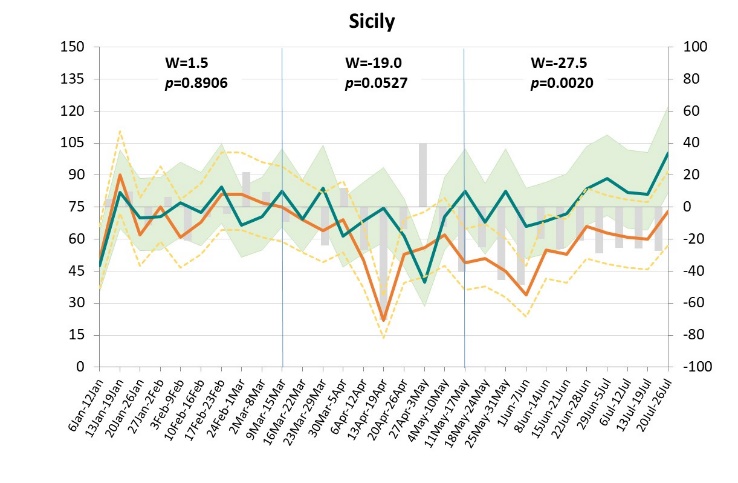 | |
| 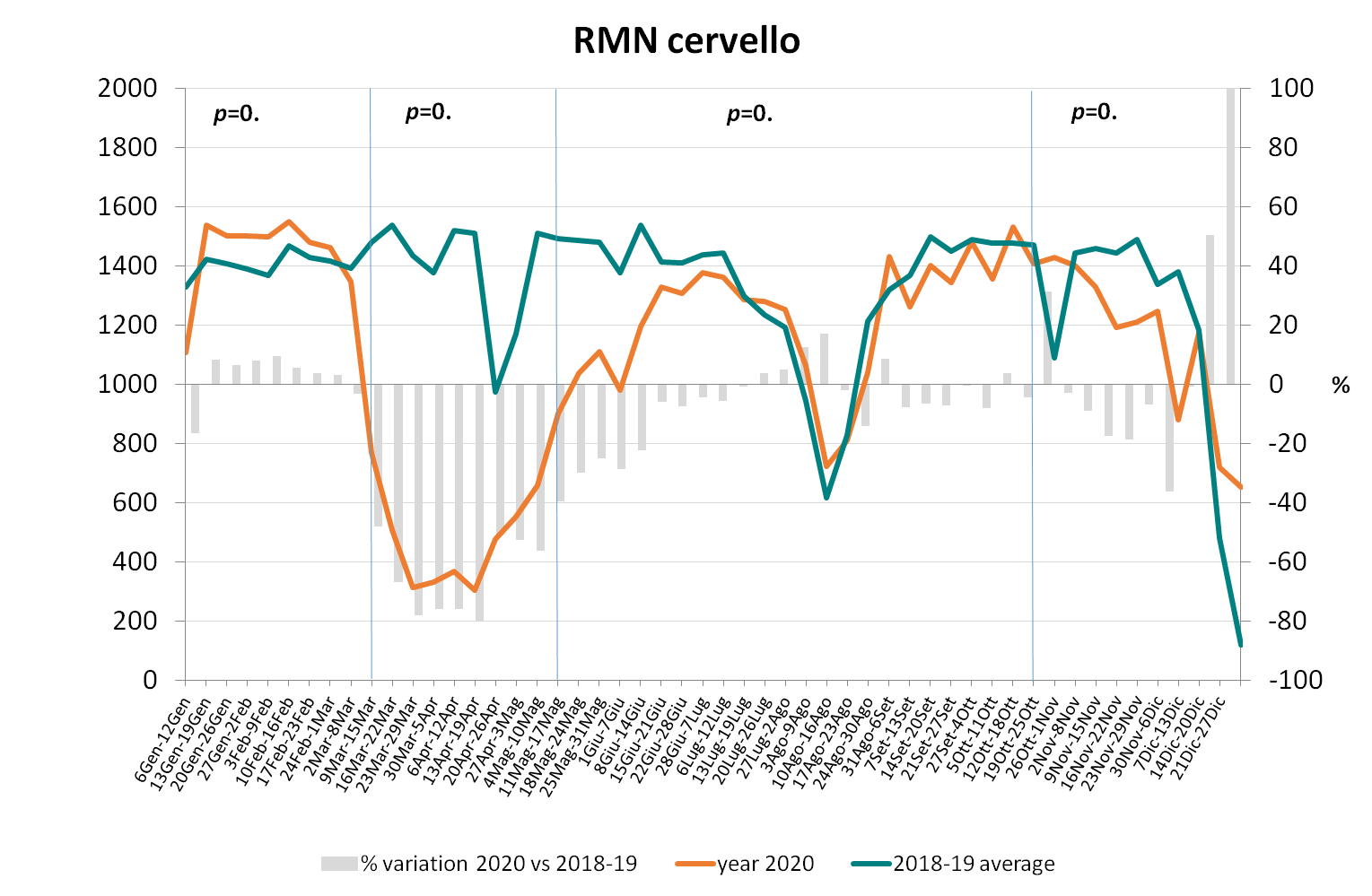 | |

* pre-lockdown, lockdown (9/3-17/5), post-lockdown – W values and p-values from the paired-sample Wilcoxon test for comparisons within each sub-period

**Figure S2d. Surgery for malignant neoplasm of prostate: volumes by region and sub-period***

Weekly trend of indicators (left axis) and percent variations (right axis) – Jan-Jul 2020 vs. 2018-19 average

| 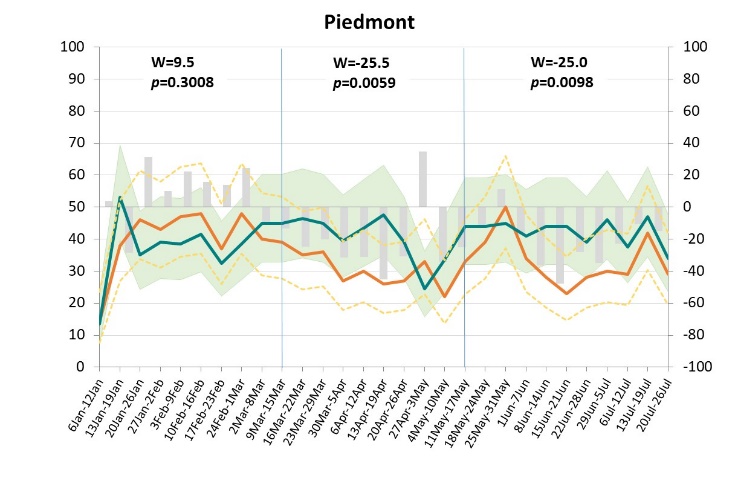 | 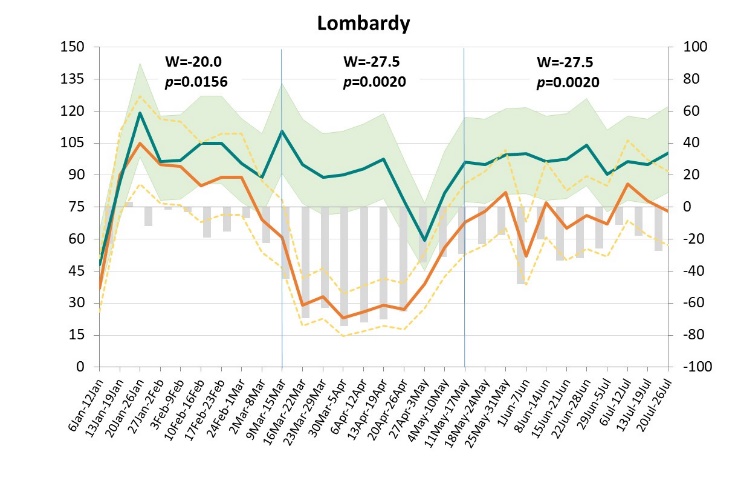 |
| --- | --- |
| 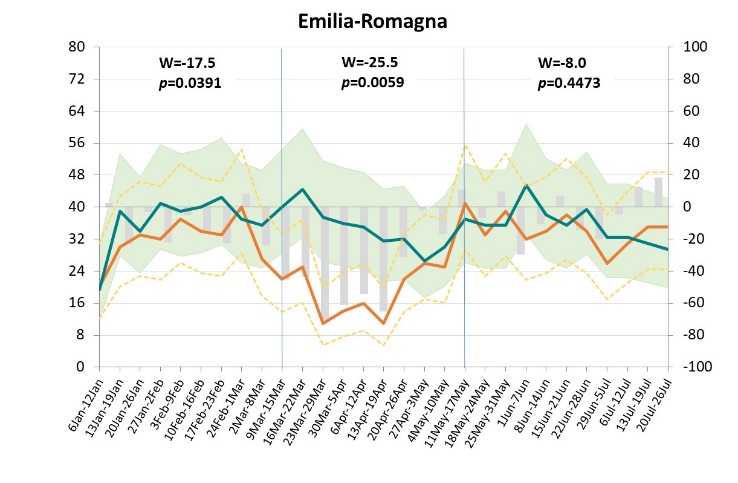 | 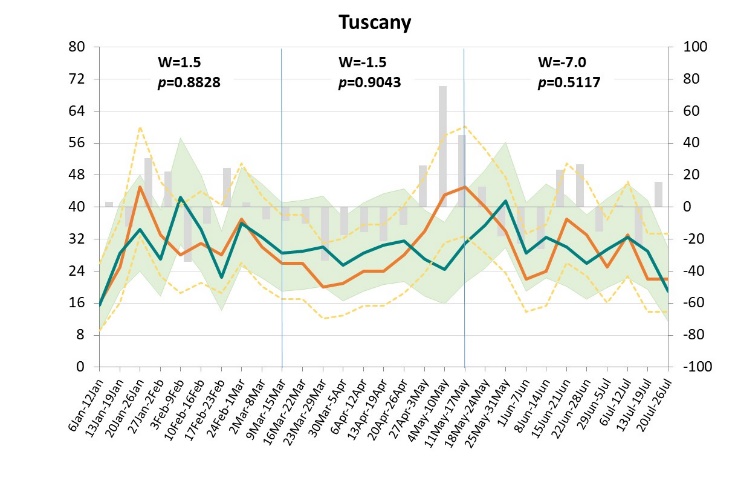 |
| 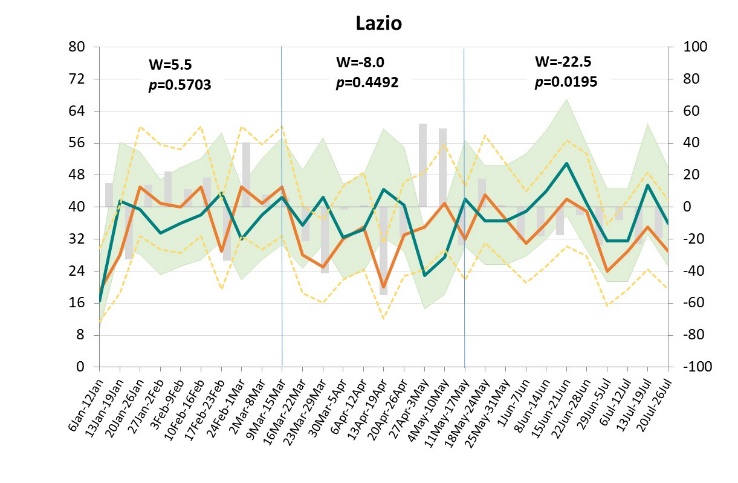 | 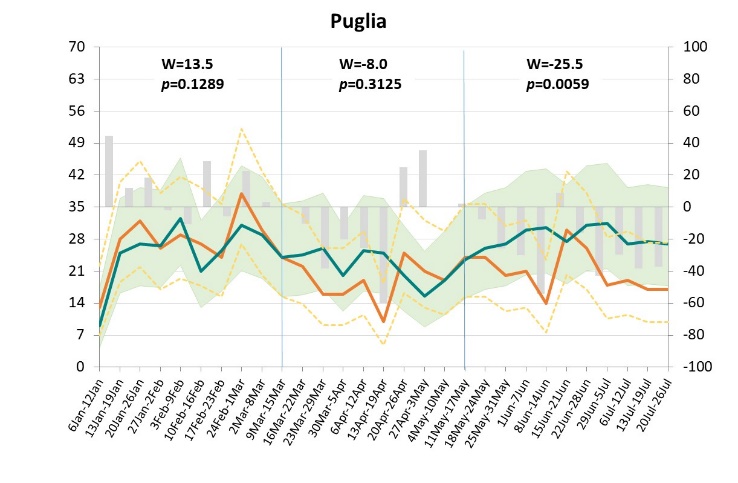 |
| 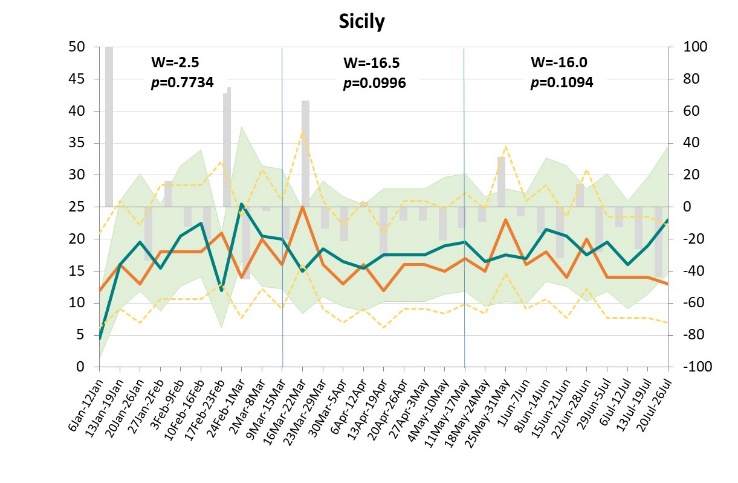 | |
| 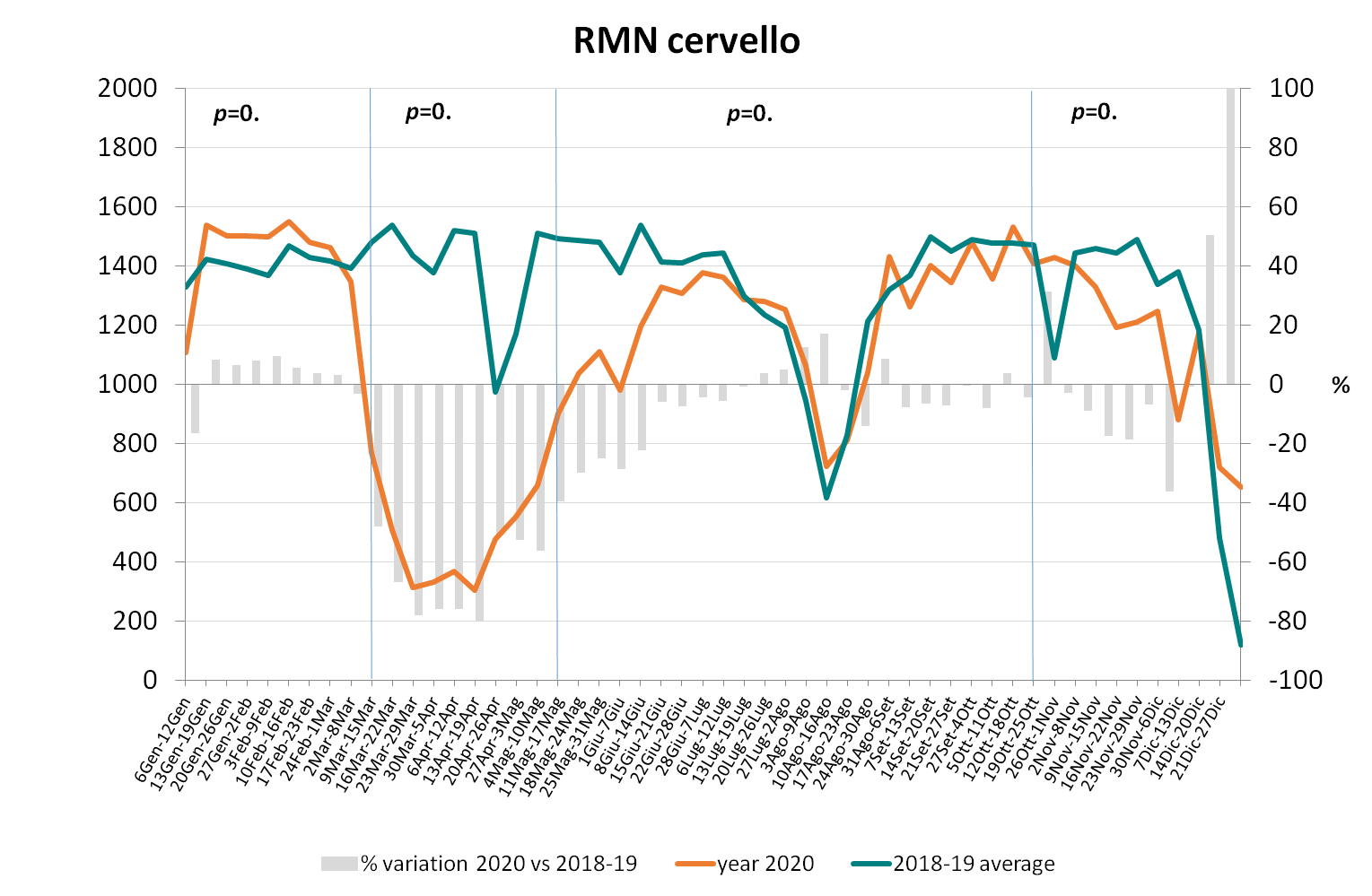 | |

* pre-lockdown, lockdown (9/3-17/5), post-lockdown – W values and p-values from the paired-sample Wilcoxon test for comparisons within each sub-period

**Figure S3a. Hospitalizations for femoral neck fracture (elderly): volumes by region and sub-period***

Weekly trend of indicators (left axis) and percent variations (right axis) – Jan-Jul 2020 vs. 2018-19 average

| 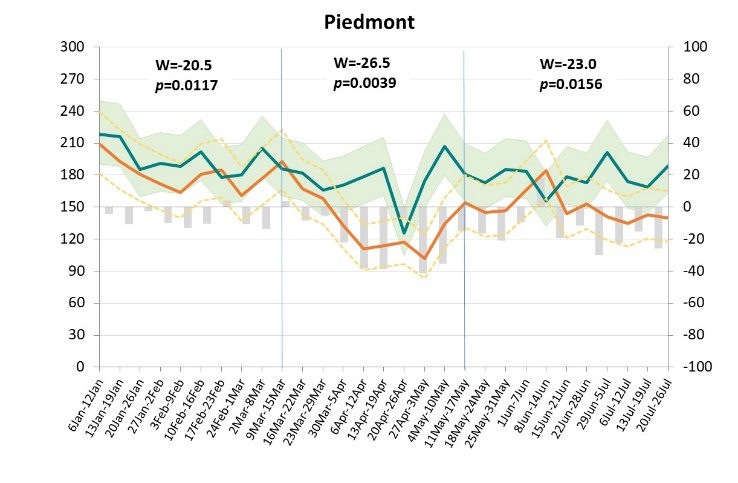 | 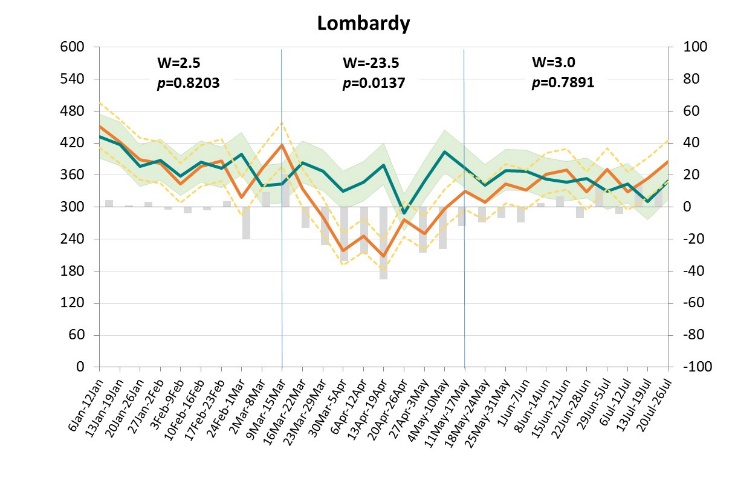 |
| --- | --- |
| 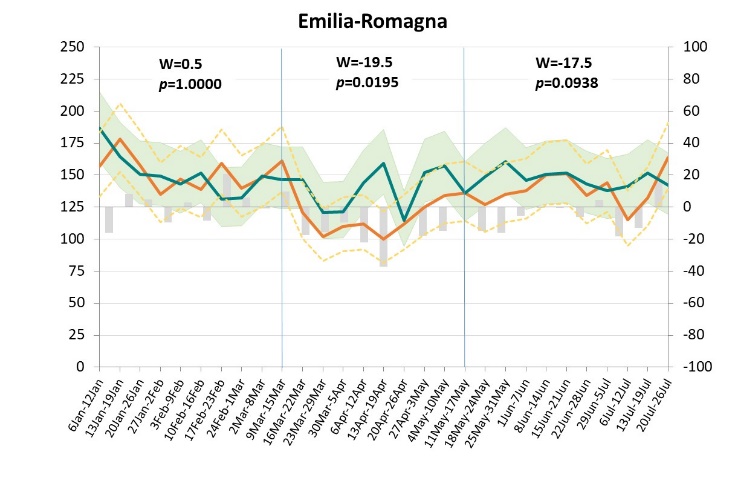 | 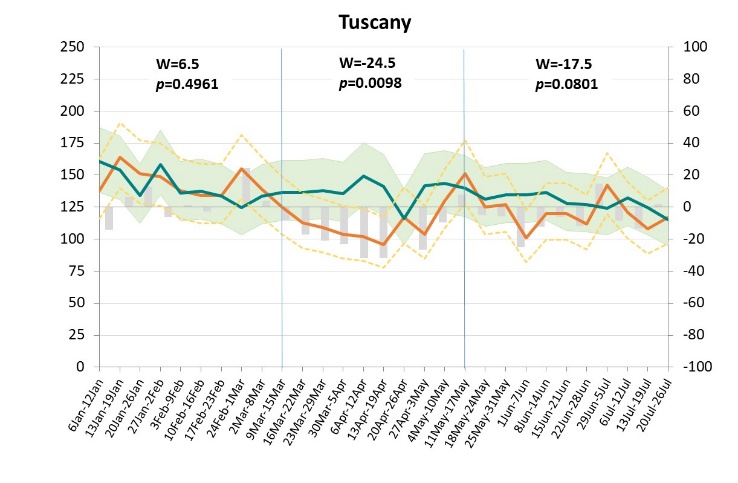 |
| 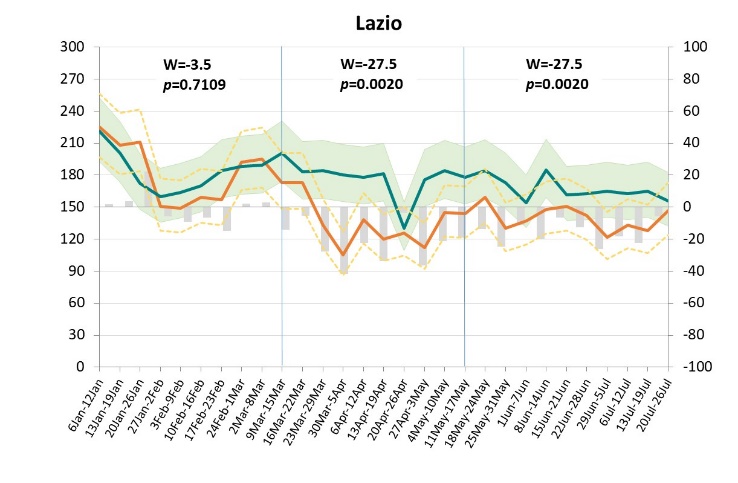 | 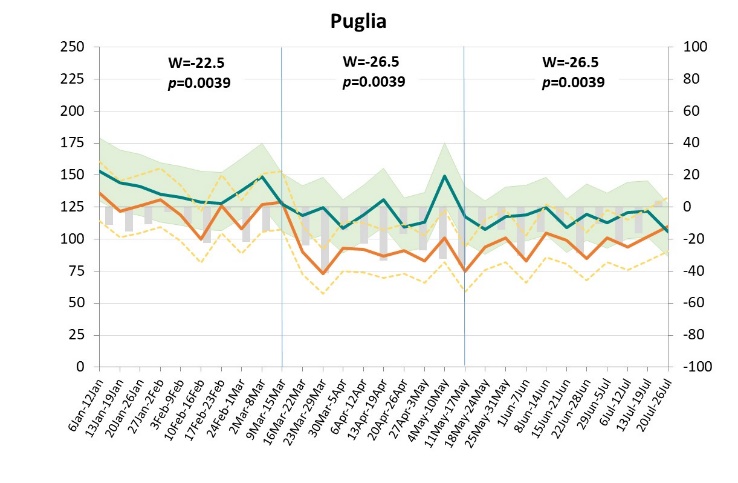 |
| 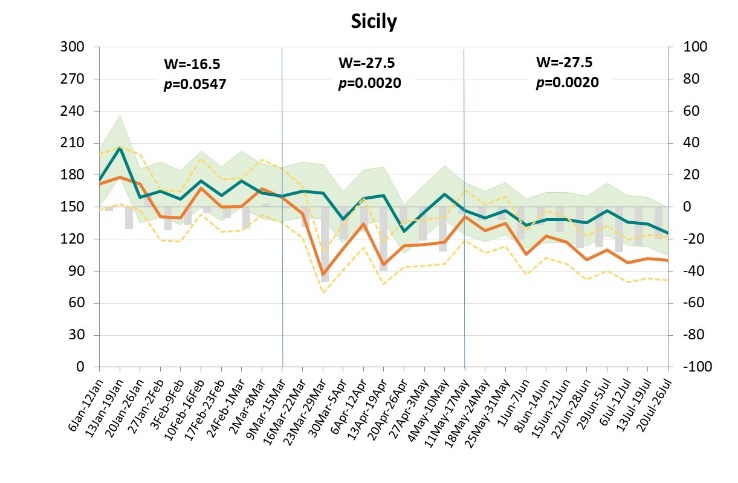 | |
| 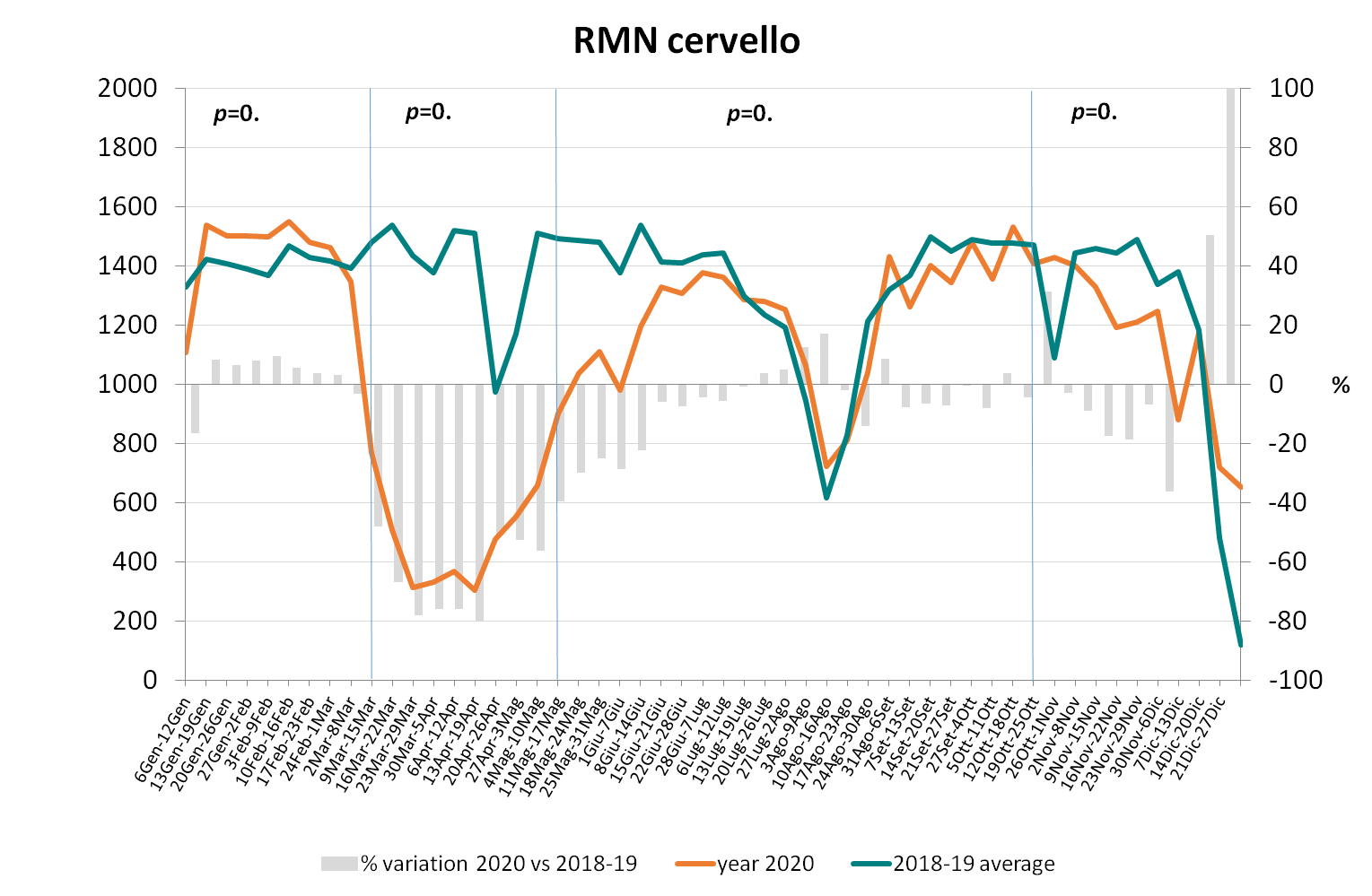 | |

* pre-lockdown, lockdown (9/3-17/5), post-lockdown – W values and p-values from the paired-sample Wilcoxon test for comparisons within each sub-period

**Figure S3b. Surgery within 2 days in femoral neck fracture (elderly): performance by region and sub-period***

Weekly trend of indicators (left axis) and percent variations (right axis) – Jan-Jul 2020 vs. 2018-19 average

| 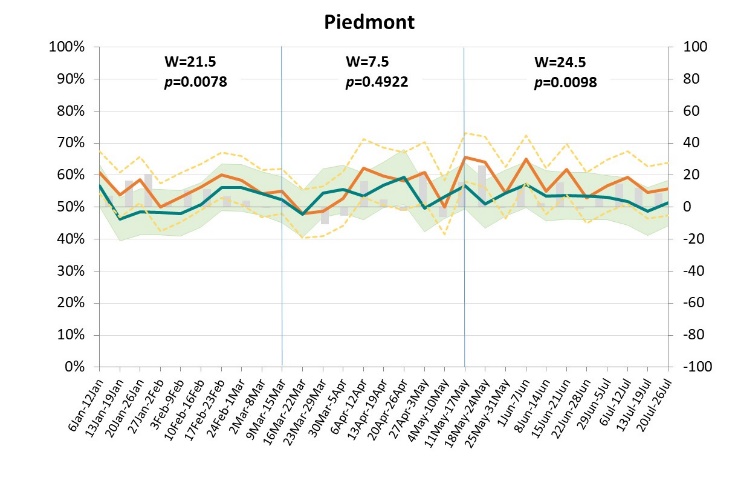 | 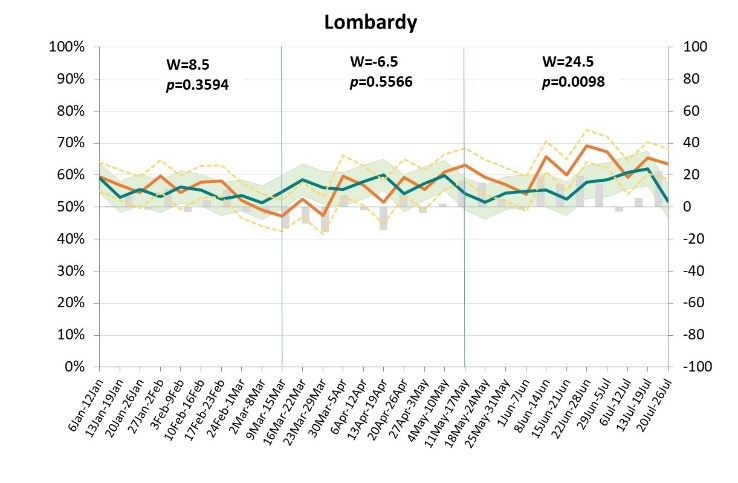 |
| --- | --- |
| 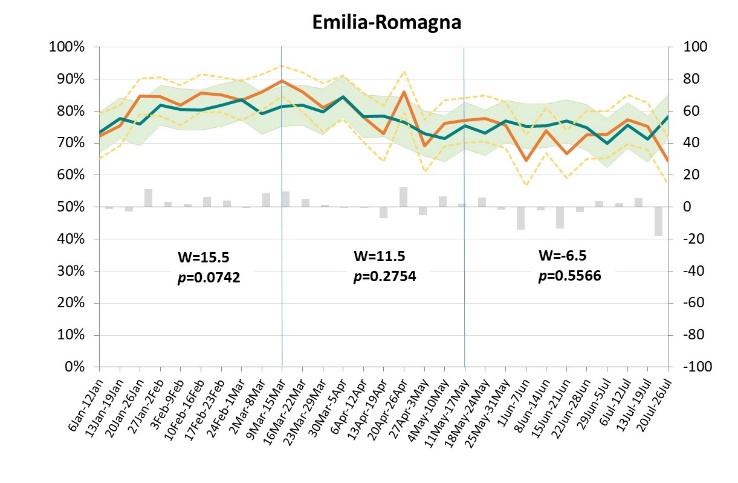 | 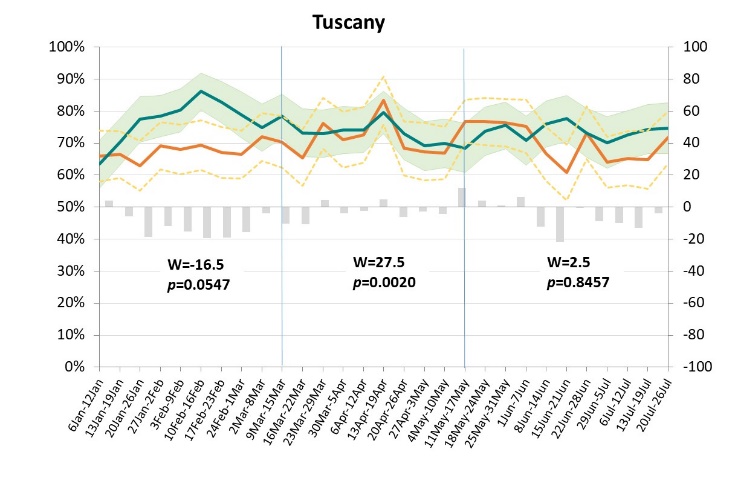 |
| 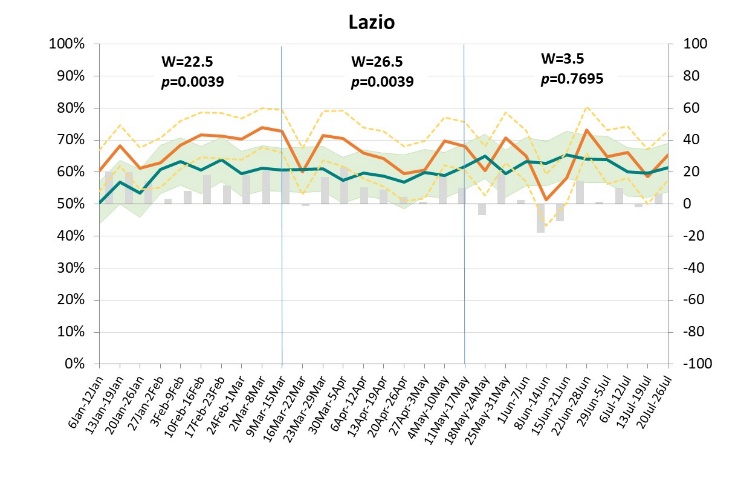 | 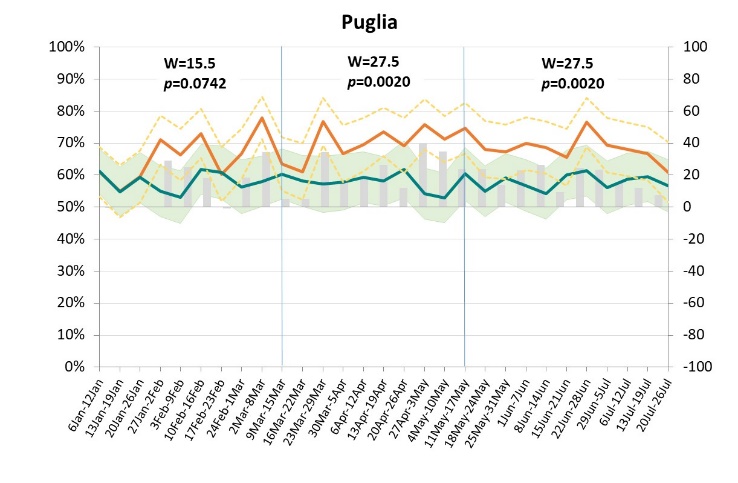 |
| 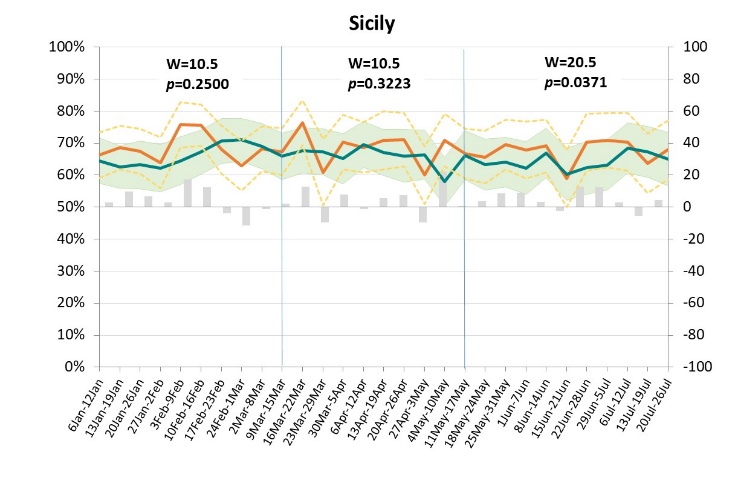 | |
| 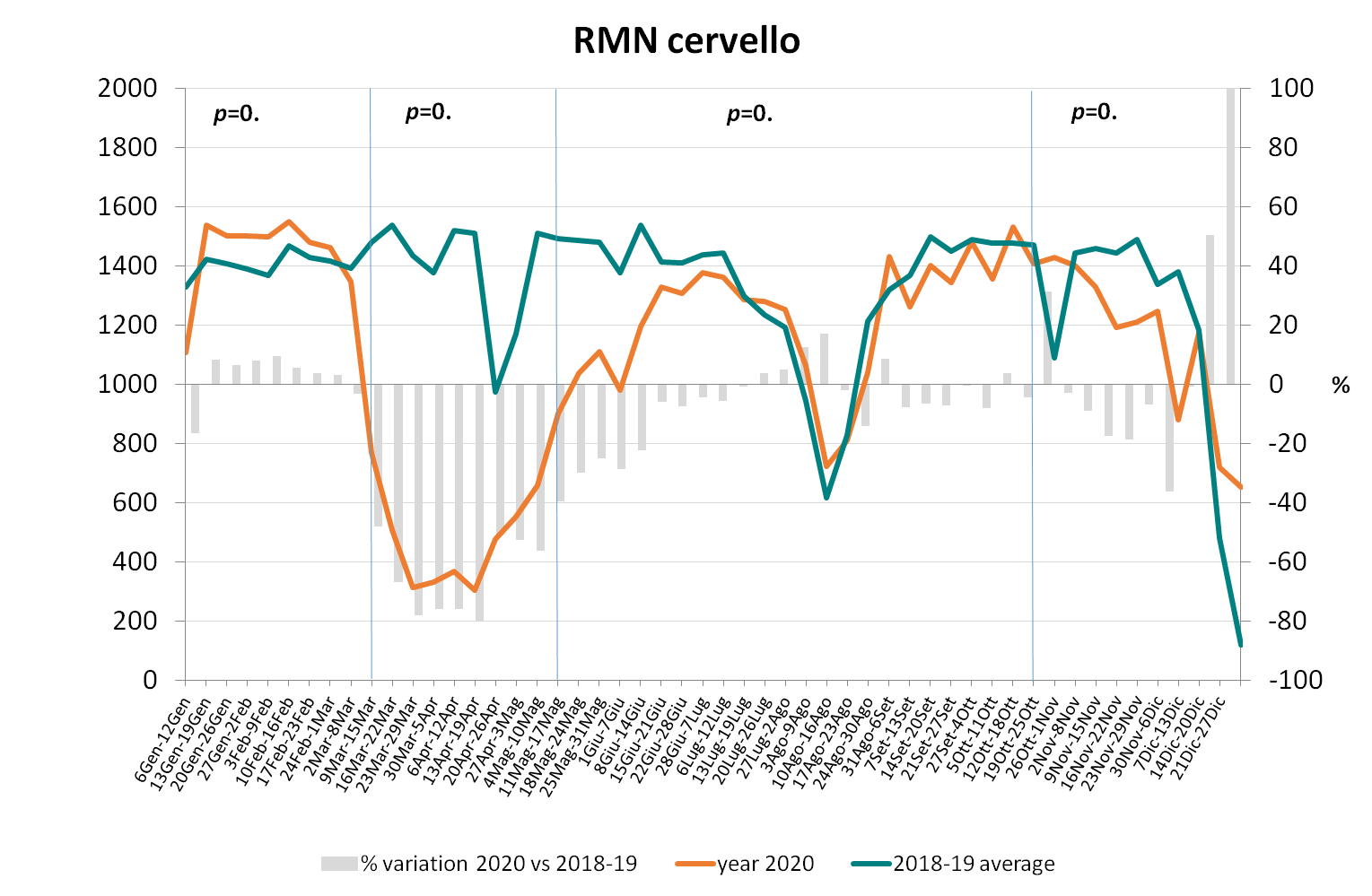 | |

* pre-lockdown, lockdown (9/3-17/5), post-lockdown – W values and p-values from the paired-sample Wilcoxon test for comparisons within each sub-period

**Figure S3c. Hip replacement surgery: volumes by region and sub-period***

Weekly trend of indicators (left axis) and percent variations (right axis) – Jan-Jul 2020 vs. 2018-19 average

| 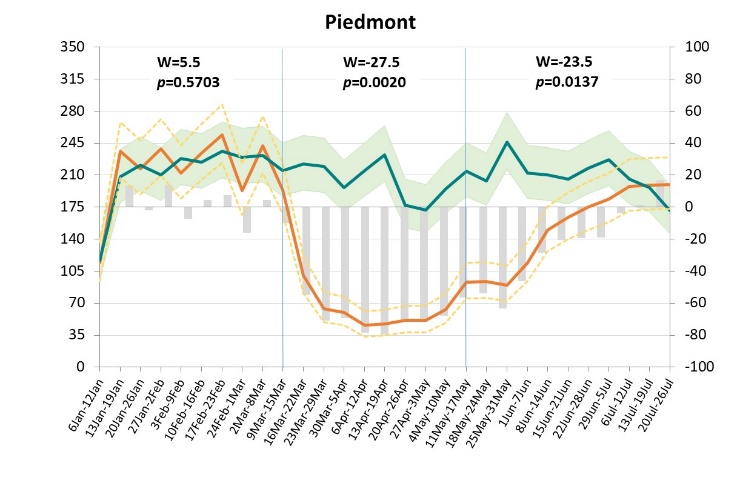 | 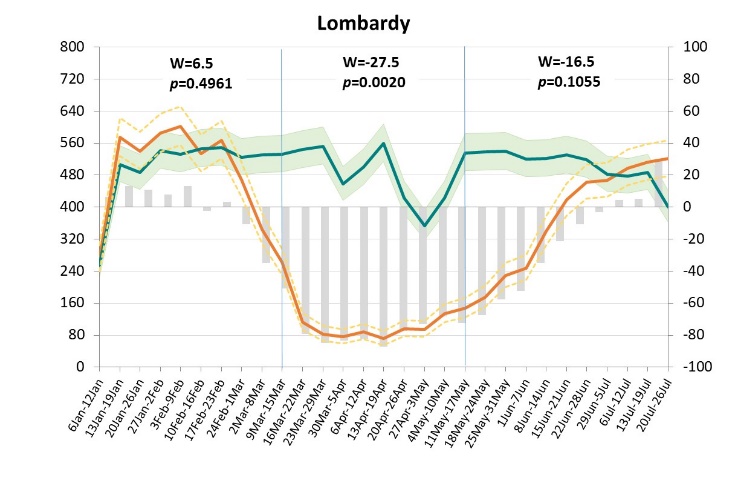 |
| --- | --- |
| 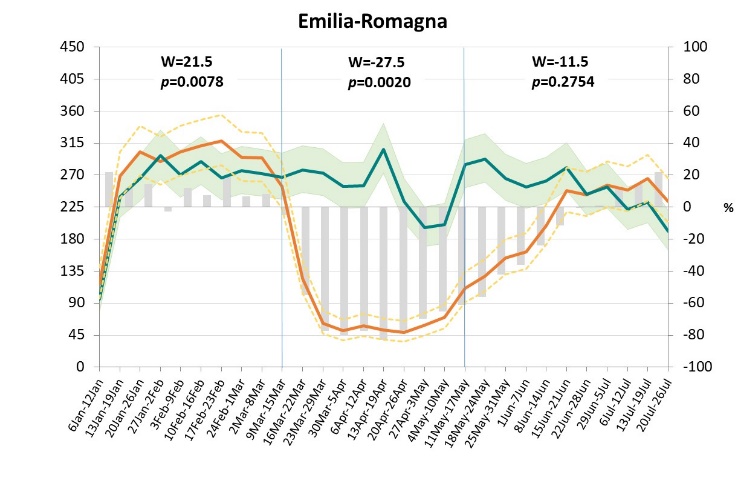 | 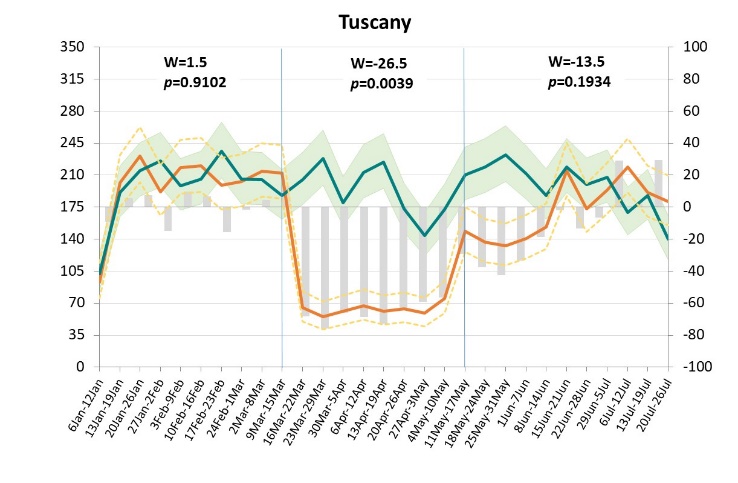 |
| 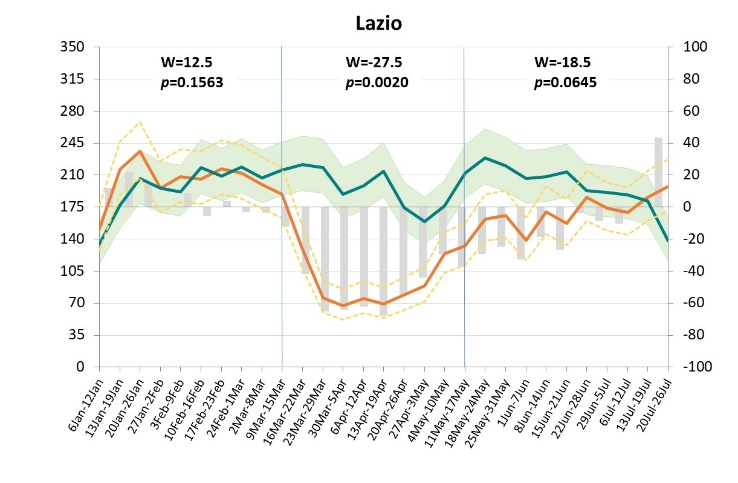 | 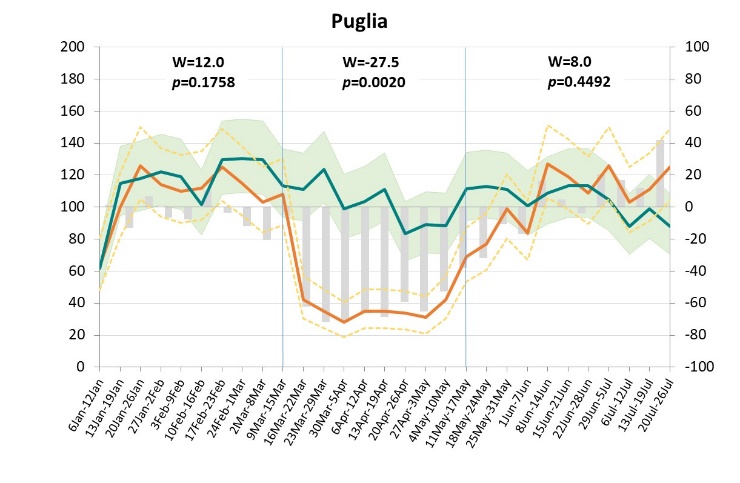 |
| 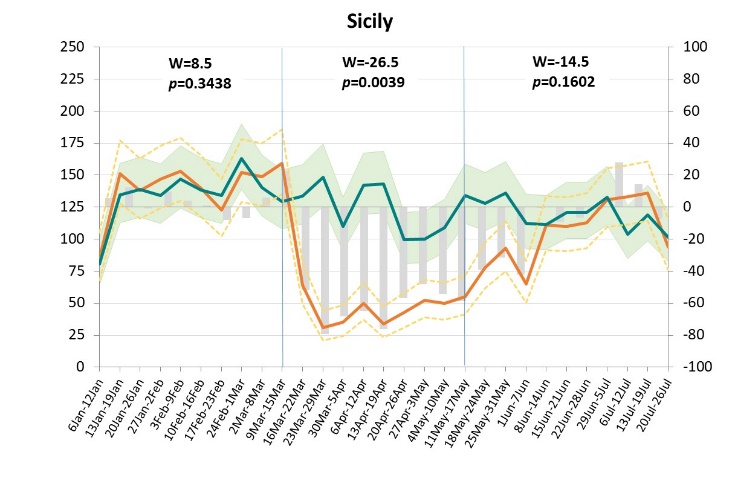 | |
| 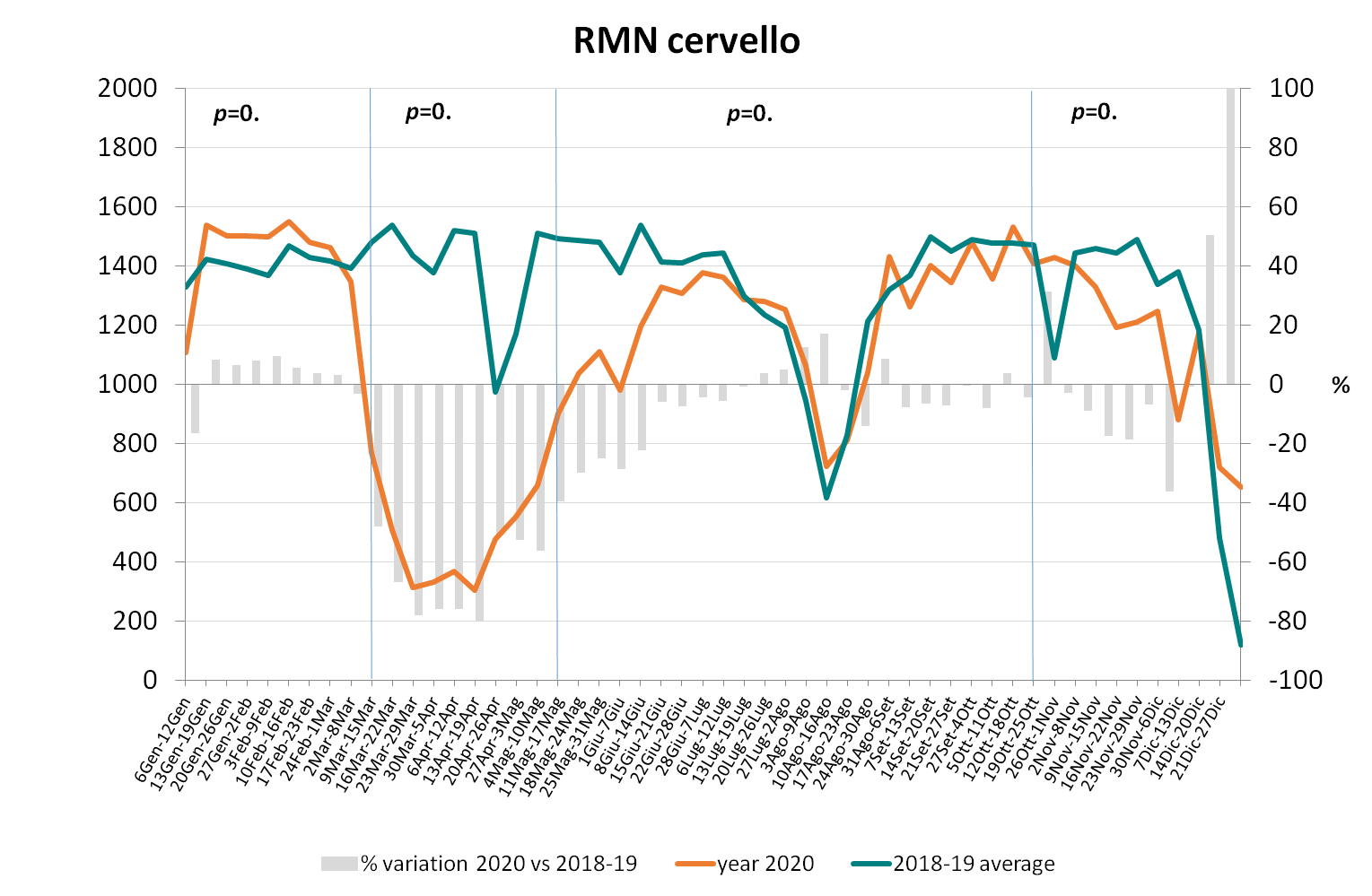 | |

* pre-lockdown, lockdown (9/3-17/5), post-lockdown – W values and p-values from the paired-sample Wilcoxon test for comparisons within each sub-period

**Figure S3d. Knee replacement surgery: volumes by region and sub-period***

Weekly trend of indicators (left axis) and percent variations (right axis) – Jan-Jul 2020 vs. 2018-19 average

| 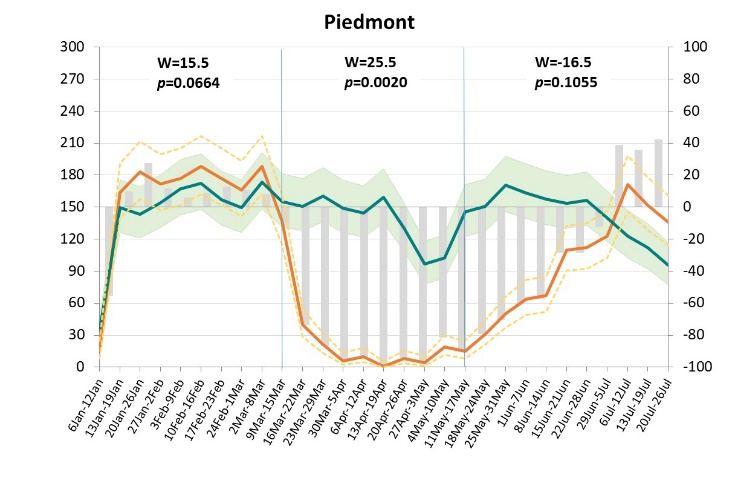 | 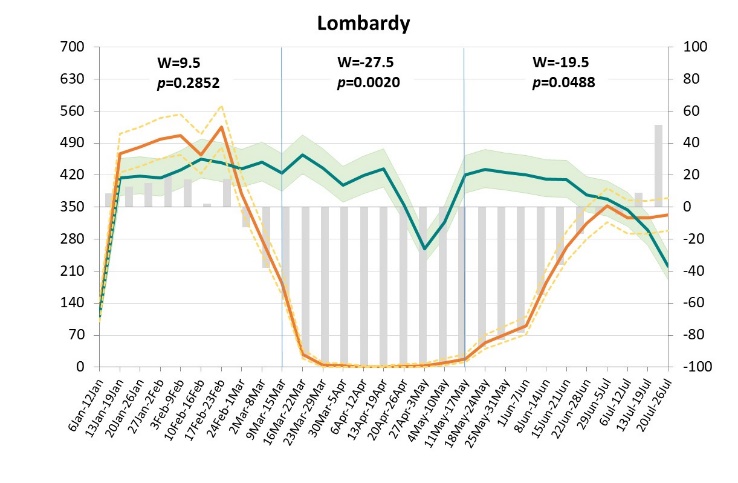 |
| --- | --- |
| 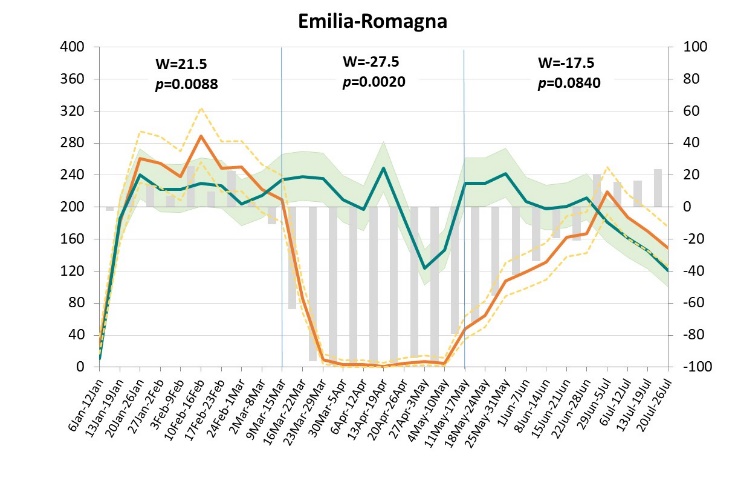 | 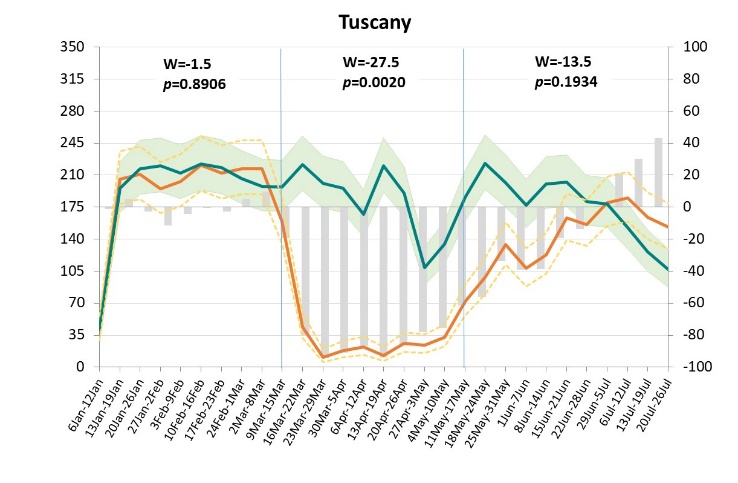 |
| 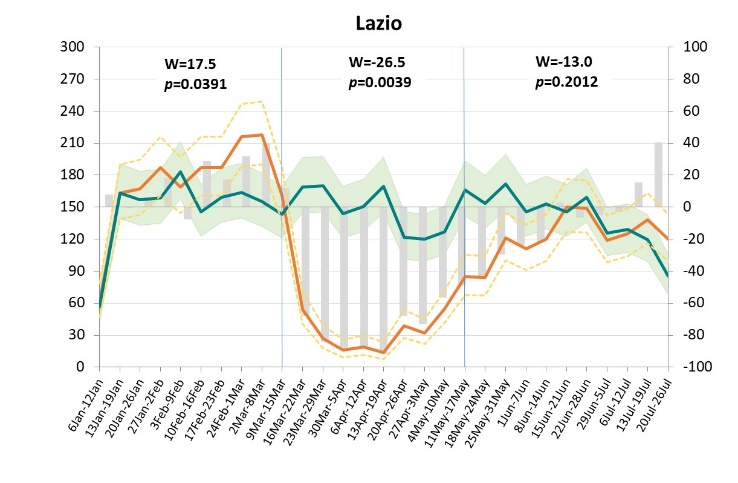 | 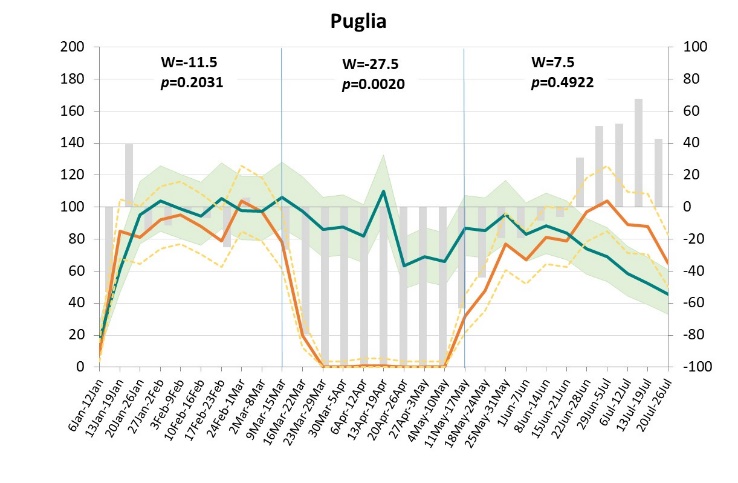 |
| 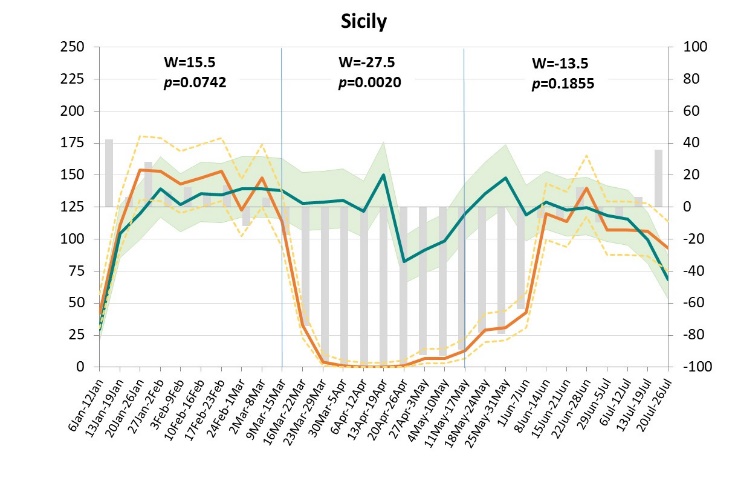 | |
| 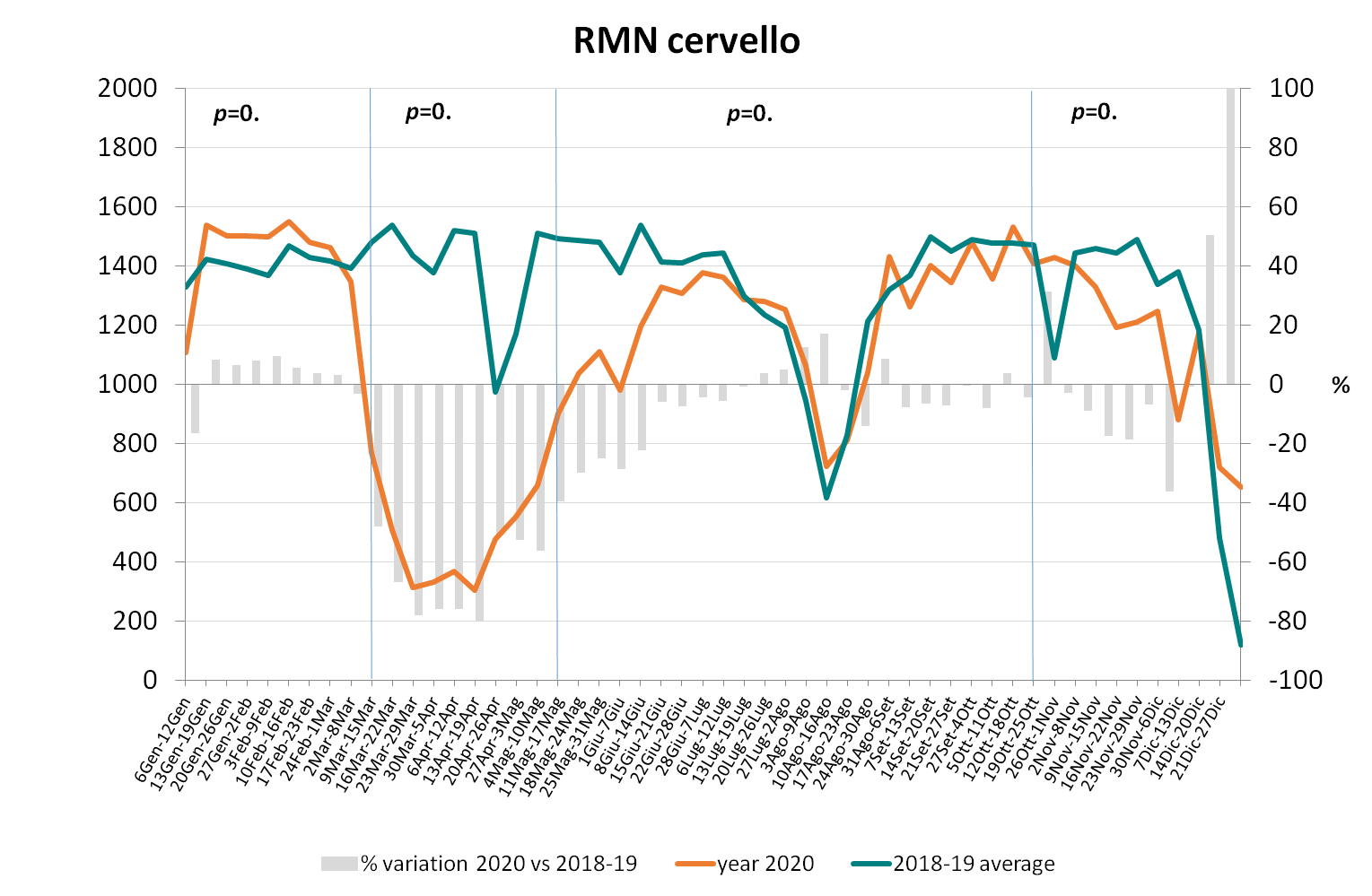 | |

* pre-lockdown, lockdown (9/3-17/5), post-lockdown – W values and p-values from the paired-sample Wilcoxon test for comparisons within each sub-period
